# Overview of the Metabolite Composition and Antioxidant Capacity of Seven Major and Minor Cereal Crops and Their Milling Fractions

**DOI:** 10.1021/acs.jafc.4c01312

**Published:** 2024-05-28

**Authors:** Luciana Ribeiro da Silva Lima, Millena C. Barros Santos, Paulo Wender P. Gomes, Álvaro Fernández-Ochoa, Mariana Simões Larraz Ferreira

**Affiliations:** † Laboratory of Bioactives, Food and Nutrition Graduate Program (PPGAN), Federal University of the State of Rio de Janeiro (UNIRIO), Rio de Janeiro 22290-240, Brazil; ‡ Center of Innovation in Mass Spectrometry, Laboratory of Protein Biochemistry, UNIRIO, Rio de Janeiro 22290-240, Brazil; § Bordeaux Metabolome-MetaboHUB, INRAE Bordeaux Nouvelle-Aquitaine, UMR1332 BFP, Villenave d’Ornon 33882, France; ∥ Collaborative Mass Spectrometry Innovation Center, Skaggs School of Pharmacy & Pharmaceutical Sciences, University of California San Diego, 9500 Gilman Drive, La Jolla, San Diego, California 92093-0751, United States; ⊥ Department of Analytical Chemistry, Faculty of Sciences, University of Granada, Granada 18071, Spain

**Keywords:** cereal coproducts, metabolomics, multivariate data analysis, phenolic compounds, polyphenols

## Abstract

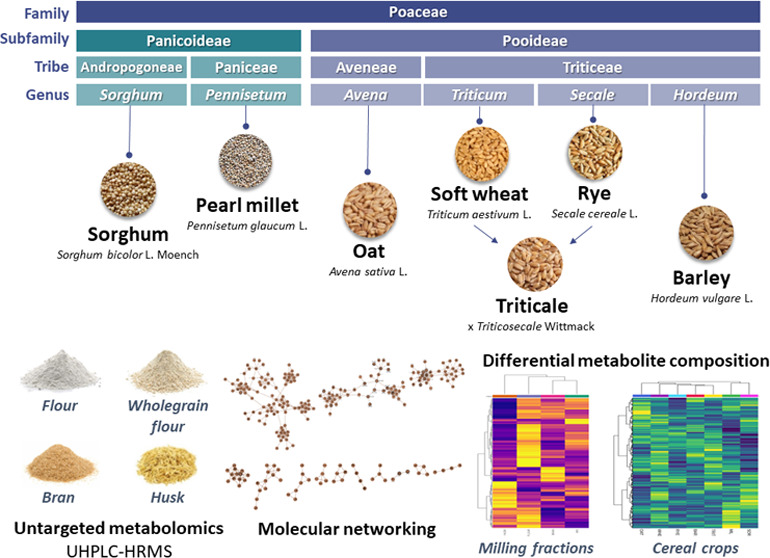

Cereal grains play an important role in human health as a source of macro- and micronutrients, besides phytochemicals. The metabolite diversity was investigated in cereal crops and their milling fractions by untargeted metabolomics ultra-high-performance liquid chromatography–tandem mass spectrometry (UHPLC–MS/MS) of 69 samples: 7 species (barley, oat, pearl millet, rye, sorghum, triticale, and wheat), 23 genotypes, and 4 milling fractions (husk, bran, flour, and wholegrain). Samples were also analyzed by *in vitro* antioxidant activity. UHPLC–MS/MS signals were processed using XCMS, and metabolite annotation was based on SIRIUS and GNPS libraries. Bran and husk showed the highest antioxidant capacity and phenolic content/diversity. The major metabolite classes were phenolic acids, flavonoids, fatty acyls, and organic acids. Sorghum, millet, barley, and oats showed distinct metabolite profiles, especially related to the bran fraction. Molecular networking and chemometrics provided a comprehensive insight into the metabolic profiling of cereal crops, unveiling the potential of coproducts and super cereals such as sorghum and millet as sources of polyphenols.

## 1 Introduction

Cereal grains are the main staple foods and have the potential to transform the global food system, toward healthier and sustainable products. Wheat, barley, oats, and sorghum are the main crops harvested worldwide, besides maize and rice.^1^ In the last years, sorghum and millet have emerged as cereals of the future, due to their potential to contribute to global food security since they have agronomic advantages such as lower water requirement and great resistance to high temperatures and drought.^2−4^

Cereal grains are largely consumed through a diversity of food and beverages (*e.g*., breads, breakfast cereals, pasta, pastry; fermented and nonfermented drinks, such as milk substitutes, roasted grain beverages, and alcoholic beverages) and play an important role in human health and nutrition, providing energy through macronutrients but also as source of micronutrients, including vitamins and minerals. In addition, they present a huge diversity of specialized metabolites with bioactive potential, including polyunsaturated fatty acids, terpenoids, tocopherols, and, especially, phenolic compounds.^4,5^

In cereal grains, phenolic compounds are mainly found in the outer layers (*e.g.*, husk, bran, and aleurone), found in free form or conjugated to glycosides (soluble fraction), or covalently bound to cell wall components (bound form), such as proteins and polysaccharides (*e.g.*, β-glucans in oats and barley grains; arabinoxylans in wheat and rye grains).^6^ The phenolic compounds are composed of many subclasses, such as phenolic acids (hydroxycinnamic acids and hydroxybenzoic acids), flavonoids (flavanols, flavones, flavanones, and others), tannins, stilbenes, and lignans. Ferulic acid and its dehydrodimers (*e.g.*, diferulic acids) are the main phenolic acids found in cereal samples, mostly in aleurone and pericarp layers.^7,8^ Lignans are also found in the outer layers of grains, especially in rye, wheat, triticale, and oat grains.^9^ The qualitative and quantitative grain phenolic composition varies according to the crop season, location, grain development stage, genotype, milling fraction, and food processing.^8,10−12^

The therapeutic value of cereal polyphenols is related to their chemical structure, spatial arrangement, and number of hydroxyls;^13^ it is believed these polyphenols target pathways of inflammation through their antioxidant, metal-chelating, and gene regulatory activities.^14^ Interactions with the intestinal epithelium allow the modulation of the intestinal microbiota and the barrier function.^15^

In the last years, metabolomics approaches have been employed to promote a comprehensive characterization of specialized metabolites in cereal samples.^11,16−19^ Most of the metabolomics-based studies focus on major crops, such as maize, rice, and wheat, while other crops, such as triticale, millet, and even sorghum, are still underexplored. According to the Web of Science platform, searching by “metabolomics” and the common name of cereal crops, the following results were obtained: “rice”, 1068 articles; “wheat”, 725; “maize” 508; “corn”, 269; “barley”, 263; “oat”, 93; “sorghum”, 85; “rye”, 75; “pearl millet”, 7, and “triticale”, 1.

Here, seven cereal crops and the effect of the milling/fractionation on the metabolite composition, focusing on phenolic compounds, were investigated based on an untargeted metabolomic approach using ultrahigh performance liquid chromatography coupled with high-resolution mass spectrometry (UHLPC–HRMS) and advanced bioinformatic tools. This work seems to provide, for the first time, a comparison based on untargeted metabolomics of the phytochemical diversity of seven different species of cereals, including major and minor crops and their milling fractions, at the same time. The recent developments and availability of modern bioinformatic and machine learning tools have allowed one to deeply investigate the huge complexity of the omics data and helped unravel the chemical profile of a diversity of samples.

## 2 Materials and Methods

### 2.1 Chemicals

Analytical standards and LC–MS-grade acetonitrile and methanol were sourced from Sigma-Aldrich (St. Louis, MO). Analytical standards include 4-hydroxybenzylalcohol, benzoic acid, gallic acid, 4-hydroxyxybenzaldehyde acid, pyrogallol, 4-hydroxybenzoic acid, *trans*-cinnamic acid, vanillin, 2,5-dihydroxybenzoic acid, chlorogenic acid, epicatechin, vanillic acid, *trans*-ferulic acid, caffeic acid, syringic acid, *p*-coumaric acid, rutin, hesperidin, myricetin, resveratrol, isoquercetrin, sinapic acid, quercetin, apigenin, kaempferol, flavanone, ellagic acid, 4-phenylacetic acid, 3,4-dihydroxyphenylacetic acid, 4-methoxycinnamic acid, and 2-hydroxycinnamic acid. The Milli-Q system (Millipore Corp., Milford, MA) was used to purify the water for analysis. Formic acid was purchased from Fluka (Switzerland). The other chemicals were of analytical grade.

### 2.2 Plant Material and Sample Preparation

Seven cereal species were analyzed in this work, comprising twenty-three genotypes and four milling fractions, totaling sixty-nine cereal samples. Samples included three oat varieties, three rye varieties, three barley varieties, five soft wheat varieties, two pearl millet varieties, three triticale varieties and four sorghum varieties. All of the cereal grains were harvested in Brazil. The four cereal milling fractions include bran, wholegrain flour, refined flour, and husk, depending on the grain. The complete sample data description can be found in Table 1 (*e.g.*, species, genotypes, crop season, and location). Due to the specific morphological characteristics of each cereal grain, refined flour, wholegrain flour, bran, and husk fractions were obtained as follows. Wheat, barley, rye, and triticale grains were processed in a Quadrumat Senior mill (Brabender, Duisburg, Germany). Oat seeds were manually dehulled, and oat groats and husks were ground in a break mill (Break mill SM1, Brabender, Duisburg, Germany). Sorghum and millet grains were decorticated into a rice polisher (Máquinas Suzuki, Brazil), and whole grains were ground in a break mill. After the milling, the flour samples passed through a 35-mesh sieve. All samples were stored at −80 °C until analyses.

**Table 1:** Samples Description

cereal crop	subfamily	tribe/genus	specie	characteristics	genotype	code	location	crop season	obtained fractions
oat	Pooideae	Aveneae/*Avena*	Avena sativa L.	husk	UPFPS Farroupilha	O1	Passo Fundo, RS	2020	H, WG
					UPFA 22-Temprana	O2			
					UPFA Fuerza	O3			
rye		Triticeae/*Secale*	Secale cereale L.	pericarp	BRS Serrano	R1	Passo Fundo, RS	2020	B, WG, F
					BRS Progresso	R2			
					BR 1	R3			
barley		Triticeae/*Hordeum*	Hordeum vulgare L.	husk	BRS Brau	B1	Passo Fundo, RS	2020	H, B, WG, F
					BRS Korbel	B2			
					BRS Elis	B3			
soft wheat		Triticeae/*Triticum*	Triticum aestivum L.	pericarp	BRS Guaraim	W1	Passo Fundo, RS	2020	B, WG, F
					BRS Marcante	W2			
					BRS Reponte	W3			
					Ametista	W4	Coxilha, RS		
					TBIO Toruk	W5			
triticale		Triticeae^a^	x *Triticosecale* Wittmack	pericarp	BRS Resoluto	T1	Passo Fundo, RS	2020	B, WG, F
					BRS Minotauro	T2			
					BRS Ulisses	T3			
pearl millet		Paniceae/*Pennisetum*	Pennisetum glaucum L.	pericarp	ADR 9070	M1	Rondonópolis, MT	2018	B, WG, F
					BRS 1502	M2	Sete Lagoas, MG		
				light brown pericarp, pigmented testa, with tannins	BRS 305	S1		2020	
sorghum	panicoideae	Andropogoneae/*Sorghum*	Sorghum bicolor L. *Moench*	red pericarp, tannin-free brown pericarp, pigmented testa, with tannins	BRS 310	S2	Sete Lagoas, MG	2017	B, WG, F
					SC 319	S3		2018	
				white pericarp, tannin-free	CMSXS 180	S4		2020	

a Triticale is a hybrid grain produced from wheat and rye grains. H = husk; WG = wholegrain flour; B = bran; and F = flour. Oat was provided by the Universidade de Passo Fundo; barley, rye, triticale, and wheat grains “BRS Guaraim,” “BRS Marcante,” and “BRS Reponte” were provided by EMBRAPA Trigo; wheat “Ametista” was provided by OR Sementes; wheat “TBIO Toruk” was provided by Biotrigo Genética; sorghum grains and pearl millet “BRS1502” were provided by EMBRAPA Milho e Sorgo; and Pearl millet “ADR 9070” was provided by ATTO Sementes.

### 2.3 Proximate Composition and Colorimetric Analysis

The moisture and ash contents were carried out according to the standard methods AACCI 44–15.02 and AACCI 08–01.01,^20^ respectively. The brightness (L*; from black (0) to white (100)), redness coordinate (a*; from green (−) to red (+)), yellowness coordinate (b*; from blue (−) to yellow (+)); hue angle (*h*_ab_), and Chroma (C*) were determined using a reflectance colorimeter (CM-5, Konica Minolta, Japan). All analyses were performed in triplicate.

### 2.4 Metabolite Extraction

The extraction of metabolites was performed as previously described.^10^ Briefly, free extracts (FPC) were generated from 70 mg of sample to which 1.5 mL of ethanol was added, stirred for 10 min (200 rpm, 25 °C), and centrifuged for 10 min (5000*g*, 25 °C). Supernatants were pooled, and the extraction was repeated one more time. To release bound compounds from cereal samples, alkaline and acid hydrolyses were applied to the residue from the ethanolic extraction. Then, bound extracts were centrifuged for 5 min at 2000*g*. Supernatants were placed in a new tube and added of ethyl acetate to recover the bound compounds (BPC). After centrifugation for 5 min (10,000*g*, 10 °C), supernatants were combined, and this step was repeated 3 times. Free and bound extracts were evaporated (Savant SpeedVac Concentrator, Thermo Fisher Scientific), redissolved in a solution of ultrapure water, methanol, and acetonitrile (93:5:2, v/v), and filtered (0.22 μm, hydrophilic PTFE, Analytica). Vials were stored at −80 °C until analyses. The extraction was performed in triplicate.

### 2.5 Estimation of the Total Phenolic Content and Antioxidant Capacity of Cereal Samples

#### 2.5.1 Total Phenolic Content Estimation

The total phenolic content was calculated by estimating the total reducing capacity of the extracts using the Folin-Ciocalteu reagent based on the classical method^21^ adapted to 96-well microplates.^19^ The absorbance was read at 750 nm on a microplate reader (FlexStation III, Molecular Devices, San Jose, California). Gallic acid was used as the standard (0–250 μg/mL; *R*^2^ = 0,999). Results were expressed as milligrams of gallic acid equivalents (GAE) per gram of sample on dry weight (dw) (mg GAE/100 g dw).

#### 2.5.2 Radical Scavenging Activity Assays

The scavenging activity of DPPH radical (2,2-diphenyl-1-picrylhydrazyl) was measured as previously described.^22^ Extracts (20 μL) were combined with 280 μL of a DPPH solution (32 μg/mL). After incubation (30 min, 25 °C), an absorbance reading was made at 517 nm. The standard curve was prepared with Trolox (6-hydroxy 2,5,7,8-tetramethylchroman-2-carboxylic acid; 0–200 μg/mL; *R*^2^ = 0,999). Results were expressed as μmol of Trolox equivalents (TE) per gram of sample on dry weight (dw) (μmol TE/g dw).

ABTS (2,2′-azino-bis(3-ethylbenzothiazoline-6-sulfonic acid)) analysis was carried out as described by Brito et al.^22^ Sample extracts were combined with 280 μL of an ABTS solution. The reading was taken at 734 nm after incubation (20 min). Trolox solution was used as a standard for the calibration curve (0–125 μg/mL; *R*^2^ = 0,999), and results were expressed as μmol of TE per g of sample on dry weight (dw) (μmol TE/g dw).

#### 2.5.3 Ferric-Reducing Antioxidant Power Assay

The determination of ferric-reducing antioxidant power (FRAP) was performed as previously described.^23^ Briefly, sample extracts (20 μL) were added to the FRAP working solution (265 μL) and ultrapure water (15 μL). Absorbance was measured at 595 nm after incubation per 30 min at 37 °C. Trolox solution (0,25 mg/mL) was used as standard for the calibration curve (0–20 μg/mL; *R*^2^ = 0,999), and results were expressed as μmol TE per g of sample on a dry weight (μmol TE/g dw).

### 2.6 Metabolomics and Bioinformatics Analysis

#### 2.6.1 Untargeted Metabolomic Profiling Analysis Based on UHPLC–HRMS

The metabolomics analysis was performed on an Acquity UPLC (Waters Corporation, Milford, MA) combined with a Xevo G2-S-Q-TOF mass spectrometer (Waters Corporation, Manchester, U.K.) equipped with an electrospray ion source operating in negative mode. Samples (5 μL) were analyzed using a column HSS T3 C18 (100 mm × 2.1 mm, 1.8 μm particle size, Waters). The flow rate was set to 0.3 mL/min, and the elution was carried out using ultrapure water and 5 mM ammonium formate (solvent A) and acetonitrile (solvent B), both acidified with 0.3% formic acid. The following gradient was applied: 0–0.5 min, 3% B; 0.5–11.8 min, 3–50% B; 11.8–12.3 min, 50–85% B; 12.3–14.7 min, 85–100% B; 14.7–16–26.2 min, 100% B; 16.2–16/7 min, 100–3% B; and last 16.7–18 min, 3% B to stabilize the system for subsequent injection. The temperatures of the column and autosampler were 30 and 8 °C, respectively. The mass spectra of data-dependent acquisition experiments were acquired according to the method previously described,^24^ with minor modifications. Data were acquired in centroid format. The following settings were applied: mass range from *m*/*z* 100 to 1200 Da; source temperature at 120 °C; cone gas flow of 50 L/h; capillary and cone voltages of 2.0 kV and 30 V, respectively; and desolvation gas (nitrogen) and temperature were set at 800 L/h and 450 °C, respectively. The number of ions selected was set to 5 (Top5 experiment); normalized collision energies of 10, 20, 30, 40, and 50 eV; a scan rate of 0.1 s; charge states of +1, + 2, and + 3; a peak extract window of 2 Da; a tolerance of deisotope ±3 Da; and an extraction tolerance of deisotope 6 Da. The peptide leucine-enkephalin was injected continuously during the analysis for mass calibration at a 300 ng/mL concentration. The acquisition was carried out in analytical batches with a maximum of 24 h of instrument time, and the instrument was calibrated before each batch. The sequence injection was randomized. Quality control samples (pooled QC, prepared by pooling equal aliquots from all extracts) were injected at the beginning of each batch and every 10 samples using the same analytical method.

#### 2.6.2 UHPLC–HRMS Data Processing

**Figure 1:**
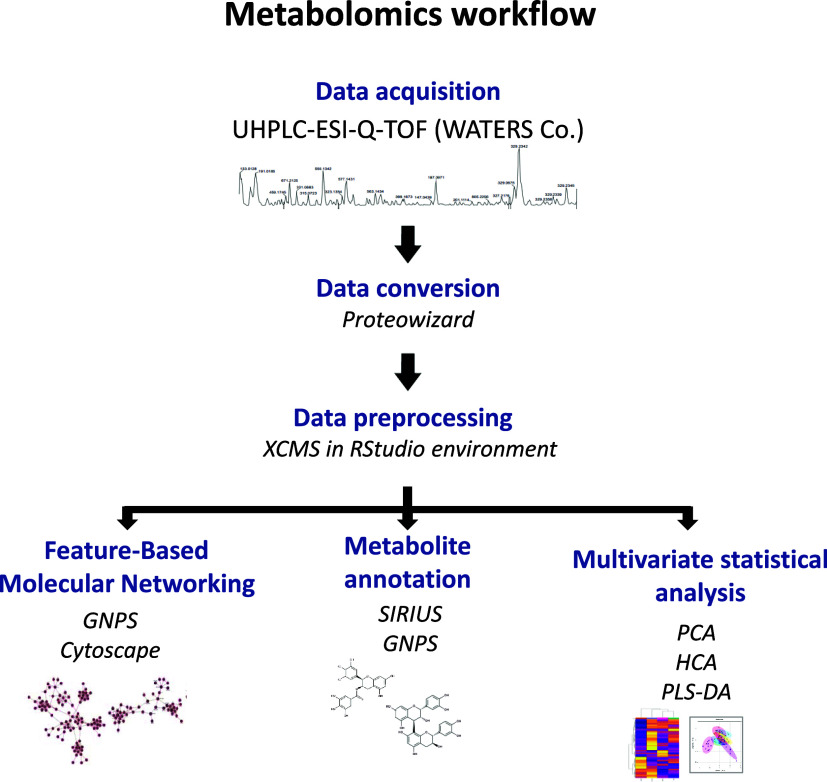
Metabolomics workflow applied in this study.

A resume of the workflow followed in this work is displayed in Figure 1. The raw data files were converted into mzML format using MSConvert tool v.3.0.21.^25^ The pooled QC samples injected at the beginning of each batch to equilibrate the column were not considered in the data processing. The mzML files were preprocessed using the RStudio environment with IPO (v 1.26.0),^26^ XCMS (v. 3.22.0),^27^ and Notame (v. 0.2.1)^28^ R packages. The IPO package was used to optimize the XCMS parameters of the peak picking, retention time alignment, and grouping steps. The optimized parameters that were used to process the sample data were as follows: peak picking (“centwave” method; (peakwidth = c(5, 25), ppm = 30, mzdiff = −0.0109, snthresh = 10, noise = 100, prefilter = c(3,100)); retention time correction (“orbwarp” method; gapInit = 0.372, gapExtend = 1.716, response = 28.16, binSize = 1)) and peak grouping (bw = 3, binSize = 0.02444). The notame package was used both to correct the drift (correct_drift function) and batch effects (normalize_batches function) based on the pooled QCs. In addition, it was also used to filter out those signals of poor quality for different reasons (low presence, contaminants, low RSD) after normalization using the following function, respectively: flag_detection (qc_limit = 0.7, group_limit = 0.75), flag_quality (RSD < 0.3 and D-ratio < 0.75), and flag_contaminants (flag_thresh = 0.25). The resulting data set after applying these normalization and filtering steps was used for statistical analysis in metaboanalyst.

Moreover, focusing on the FBMN analysis, after the peak grouping step at the XCMS processing (last function), the.mgf file containing only MS^2^ spectra was generated with the customized function “formatSpectraForGNPS” (GitHub repository https://github.com/jorainer/xcms-gnps-tools) and added the quantification table in.csv format. This.mgf file was also imported into SIRIUS 5^29^ for *in silico* annotation, visualization of the fragmentation pattern and relative intensity of each *m*/*z*, prediction of the molecular formula, and metabolite classification by the CANOPUS tool. The metabolite annotation followed the levels of identification, previously established:^30^ 1, compared to the authentic standards; 2, putatively annotation based on MS/MS spectral similarity with public libraries; 3, putatively class annotation based on the MS/MS spectral similarity to other annotated compounds and/or classified by the CANOPUS tool on SIRIUS software; and 4, unknown compounds.

#### 2.6.3 Molecular Networking

Files were submitted to Feature-Based Molecular Networking (FBMN)^31^ analysis on GNPS^32^ (https://gnps.ucsd.edu). The data was filtered by removing all MS^2^ ions within ±17 Da of the precursor *m*/*z*. MS^2^ spectra were window-filtered by choosing only the top 6 fragment ions in the ±50 Da window throughout the spectrum. Precursor and fragment ion mass tolerances were set to 0.05 Da. The edges were filtered to have a cosine score of above 0.6 and more than 6 matched peaks. The maximum size of a molecular family was set to 100, and the lowest-scoring edges were removed from molecular families until the molecular family size was below this threshold. The MS/MS data were searched against GNPS spectral libraries using the same settings as those of the input data. The minimum required to keep the match between network spectra and library spectra was a score of 0.7 and 4 matched peaks. Then, the molecular networking visualization was made using Cytoscape software version 3.10.1.^33^

### 2.7 Statistical Analysis

Results from the antioxidant assays, ash content, and colorimetry were analyzed by the one-way factor of variance (ANOVA), followed by Tukey’s Test with a level of significance of 5% (*p* < 0.05) to compare the mean values using the SPPS IBM SPSS Statistics version 28. The multivariate (PCA, PLS-DA, hierarchical clustering *via* heatmap) analyses were performed for the metabolomics data by using the web platform MetaboAnalyst 5.0 (https://www.metaboanalyst.ca/), with missing values being replaced by 1/5 of minimum positive values of their corresponding variables, log10 transformation, and Pareto-scaling. Two different PLS-DA models were performed to analyze (1) cereal crops and (2) milling fractions. Thus, it was possible to extract from the PLS-DA models the variable importance for the projection (VIP) scores to distinguish each group of samples. Relationships AMONG the ash content, colorimetry, phenolic content estimation, antioxidant capacity, and metabolite composition were studied by a linear correlation matrix (*p* < 0.05).

## 3 Results and Discussion

### 3.1 Ash Content and Colorimetric Analysis of Whole Cereals and Their Milling Fractions

The moisture content used to calculate the dry weight (dw) of all samples can be seen in Table S2. The ash content and colorimetry measurements of the flour samples are displayed in Table 2. The ash content varied from 0.59% (W5) to 1.77% (M1) and averaged 1.04% ± 0.34. Wheat, rye, and triticale flours showed the lowest ash contents and the highest values for L*, being whiter than sorghum and pearl millet flours. While the samples of pearl millet, B3 (barley), and S3 (sorghum) showed the highest ash contents. The highest values of a* and b* were found in sorghum samples with colored pericarp (S1–S3). The chroma and hue angle values varied from 5.93 to 13.72 and 67.43–87.92, respectively.

**Table 2:** Ash Content and Colorimetric Analysis of Flour Samples^a^

cereal crop	gen	ash (%)	L*	a*	b*	chroma	*h*_ab_
rye	R1	0.85 ± 0.02^cdefg^	90.68 ± 0.07^iJ^	0.69 ± 0.02^ghi^	7.24 ± 0.03^jk^	7.27 ± 0.03^jk^	84.51 ± 0.12^g^
	R2	0.89 ± 0.02^cdef^	91.60 ± 0.07^fg^	0.46 ± 0.01^ghi^	7.08 ± 0.02^kl^	7.10 ± 0.03^kl^	86.30 ± 0.09^de^
	R3	0.85 ± 0.02^cdefg^	90.88 ± 0.03^hiJ^	0.55 ± 0.01^ghi^	6.86 ± 0.03^l^	6.88 ± 0.03^l^	85.44 ± 0.06^f^
***mean rye***		***0.87 ± 0.02***	***91.05 ± 0.48***	***0.57 ± 0.12***	***7.06 ± 0.19***	***7.08 ± 0.20***	***85.42 ± 0.90***
barley	B1	0.94 ± 0.01^cde^	92.07 ± 0.03^f^	0.77 ± 0.01^fgh^	6.85 ± 0.04^l^	6.89 ± 0.04^l^	83.57 ± 0.02^h^
	B2	0.94 ± 0.02 ^cde^	93.22 ± 0.02^cd^	0.56 ± 0.01^ghi^	5.90 ± 0.05^m^	5.93 ± 0.05^m^	84.59 ± 0.08^g^
	B3	1.54 ± 0.04^ab^	90.47 ± 0.07^J^	1.11 ± 0.02^ef^	8.21 ± 0.11^h^	8.28 ± 0.11^h^	82.29 ± 0.01i
***mean barley***		***1.14 ± 0.35***	***91.92 ± 1.38***	***0.81 ± 0.28***	***6.99 ± 1.16***	***7.03 ± 1.18***	***83.48 ± 1.16***
pearl millet	M1	1.77 ± 0.02^a^	74.03 ± 0.20°	0.83 ± 0.00^fg^	11.66 ± 0.18^b^	11.69 ± 0.18^c^	85.94 ± 0.05^def^
	M2	1.45 ± 0.01^b^	71.35 ± 0.13^p^	1.25 ± 0.03^e^	13.05 ± 0.14^a^	13.11 ± 0.14^b^	84.53 ± 0.09^g^
***mean pearl millet***		***1.61 ± 0.22***	***72.69 ± 1.90***	***1.04 ± 0.30***	***12.35 ± 0.98***	***12.40 ± 1.00***	***85.23 ± 0.99***
wheat	W1	0.67 ± 0.03^efgh^	94.30 ± 0.18^a^	0.36 ± 0.03^hi^	7.53 ± 0.20^iJ^	7.53 ± 0.21^iJ^	87.44 ± 0.39^abc^
	W2	0.81 ± 0.02^cdefg^	92.66 ± 0.09^e^	0.73 ± 0.01^fgh^	10.08 ± 0.07^de^	10.11 ± 0.06^f^	85.83 ± 0.01^ef^
	W3	0.62 ± 0.11^fgh^	93.17 ± 0.07^cde^	0.61 ± 0.02^ghi^	9.81 ± 0.01^ef^	9.83 ± 0.01^f^	86.41 ± 0.08^d^
	W4	0.61 ± 0.01^fgh^	91.26 ± 0.13^gh^	0.36 ± 0.01^hi^	9.36 ± 0.12^g^	9.37 ± 0.12^g^	87.81 ± 0.06^d^
	W5	0.59 ± 0.01^gh^	93.63 ± 0.02^bc^	0.39 ± 0.01^hi^	10.60 ± 0.01^c^	10.61 ± 0.02^e^	87.92 ± 0.03^a^
***mean wheat***		***0.66 ± 0.09***	***93.01 ± 1.15***	***0.49 ± 0.17***	***9.48 ± 1.18***	***9.49 ± 1.18***	***87.08 ± 0.92***
triticale	T1	1.04 ± 0.02^cd^	91.03 ± 0.04^hi^	0.64 ± 0.01^ghi^	9.27 ± 0.02^g^	9.30 ± 0.02^g^	86.02 ± 0.03^de^
	T2	0.69 ± 0.02^efgh^	93.74 ± 0.06^b^	0.31 ± 0.01^i^	6.74 ± 0.06^l^	6.75 ± 0.06^l^	87.37 ± 0.09^bc^
	T3	0.75 ± 0.02^defg^	92.74 ± 0.07^de^	0.41 ± 0.00^hi^	7.88 ± 0.18^hi^	7.89 ± 0.18^i^	87.01 ± 0.07^c^
***mean triticale***		***0.83 ± 0.18***	***92.50 ± 1.37***	***0.45 ± 0.17***	***7.97 ± 1.27***	***7.98 ± 1.28***	***86.80 ± 0.70***
sorghum	S1	1.06 ± 0.05^c^	83.15 ± 0.41^k^	2.39 ± 0.04^d^	11.32 ± 0.16^b^	11.57 ± 0.16^cd^	78.07 ± 0.20^1^
	S2	1.06 ± 0.08^c^	77.93 ± 0.18^n^	4.94 ± 0.59^a^	12.92 ± 0.25^a^	13.72 ± 0.25^a^	70.40 ± 0.17^l^
	S3	1.41 ± 0.03^b^	79.45 ± 0.26^m^	4.33 ± 0.09^b^	10.41 ± 0.20^cd^	11.27 ± 0.21^d^	67.43 ± 0.05^m^
	S4	1.09 ± 0.07^c^	80.31 ± 0.37^l^	3.12 ± 0.07^c^	9.47 ± 0.08^fg^	9.96 ± 0.07^f^	71.79 ± 0.49^k^
***mean sorghum***		***1.14 ± 0.15***	***80.21 ± 2.19***	***3.69 ± 1.15***	***11.03 ± 1.47***	***11.63 ± 1.56***	***71.92 ± 4.49***
**mean flour**		**1.04 ± 0.34**	**86.90 ± 8.46**	**1.18 ± 1.25**	**9.15 ± 2.21**	**9.27 ± 2.32**	**83.32 ± 5.73**

a gen = genotype; L* = brightness coordinate; a* = redness coordinate; b* = yellowness coordinate; C* = chroma; *h*_ab_ = hue angle. Different letters in each column indicate significant differences (Tukey’s test, *p* < 0.05). Bold values are the mean values (mean ± SD) of cereal crops.

The ash content of wholegrain flours ranged from 1.38% (S2) to 2.81% (B3), averaging 1.90% and 77.94 of the brightness value (Table 3). Sorghum samples showed the lowest mean value of ash content, while barley and oat grains presented the highest ash content. Barley, rye, triticale, and wheat grains were the whitest wholegrain flours when compared with other whole samples. The redness coordinate was higher in sorghum, especially in the reddish sample S3 (a* value of 6.36) between whole samples. Oat grains presented the highest b* mean value (13.46).

**Table 3:** Ash Content and Colorimetric Analysis of Wholegrain Flours^a^

cereal crop	gen	ash (%)	L*	a*	b*	chroma	*h*_ab_
oat	O1	2.00 ± 0.11^bc^	80.69 ± 0.22^e^	2.46 ± 0.09^h^	12.51 ± 0.25^b^	12.75 ± 0.25^cd^	78.70 ± 0.19^cd^
	O2	2.06 ± 0.01^b^	79.48 ± 0.06^f^	3.18 ± 0.04^f^	15.03 ± 0.31^a^	15.36 ± 0.30^a^	78.05 ± 0.17^e^
	O3	2.05 ± 0.06^b^	75.82 ± 0.08^h^	2.42 ± 0.09^h^	12.84 ± 0.48^b^	13.07 ± 0.48^c^	79.33 ± 0.26^c^
***mean oat***		***2.04 ± 0.03***	***78.66 ± 2.54***	***2.69 ± 0.43***	***13.46 ± 1.37***	***13.73 ± 1.42***	***78.75 ± 0.65***
rye	R1	2.08 ± 0.04^b^	82.92 ± 0.04^d^	1.85 ± 0.07^ij^	8.51 ± 0.05^ijk^	8.71 ± 0.06^klm^	77.66 ± 0.38^e^
	R2	1.95 ± 0.09^bcd^	83.67 ± 0.23^c^	1.77 ± 0.08J^kl^	9.02 ± 0.05^hi^	9.18 ± 0.05^ijk^	86.30 ± 0.09^c^
	R3	1.72 ± 0.01^efg^	81.28 ± 0.32^e^	1.92 ± 0.01^ij^	8.69 ± 0.19^ij^	8.90 ± 0.18^jkl^	77.55 ± 0.29^e^
***mean rye***		***1.92 ± 0.19***	***82.62 ± 1.22***	***1.85 ± 0.08***	***8.74 ± 0.26***	***8.93 ± 0.23***	***78.16 ± 0.97***
barley	B1	2.08 ± 0.02^b^	84.24 ± 0.29^c^	1.80 ± 0.04^ijk^	9.52 ± 0.10^fgh^	9.69 ± 0.10^i^	83.57 ± 0.02^c^
	B2	1.96 ± 0.03^bcd^	86.16 ± 0.16^a^	1.61 ± 0.04^klm^	8.47 ± 0.15^jk^	8.63 ± 0.15^lm^	79.26 ± 0.1^c^
	B3	2.81 ± 0.04^a^	82.89 ± 0.05^d^	2.46 ± 0.07^h^	11.90 ± 0.09^d^	12.15 ± 0.10^ef^	78.33 ± 0.22^de^
***mean barley***		***2.28 ± 0.46***	***84.43 ± 1.64***	***1.96 ± 0.44***	***9.97 ± 1.76***	***10.16 ± 1.81***	***78.95 ± 0.54***
pearl millet	M1	1.96 ± 0.05^bcd^	68.60 ± 0.17^l^	1.56 ± 0.02^lm^	11.97 ± 0.17^cd^	12.07 ± 0.17^ef^	82.56 ± 0.08^a^
	M2	1.62 ± 0.14^gh^	63.88 ± 0.12^m^	2.01 ± 0.08^i^	12.89 ± 0.08^b^	13.04 ± 0.09^c^	84.54 ± 0.09^b^
***mean pearl millet***		***1.79 ± 0.24***	***66.24 ± 3.34***	***1.79 ± 0.32***	***12.43 ± 0.65***	***12.56 ± 0.69***	***81.85 ± 1.01***
wheat	W1	1.99 ± 0.01^bc^	85.46 ± 0.25^b^	1.98 ± 0.08ij	9.21 ± 0.18^gh^	9.42 ± 0.19^ij^	77.84 ± 0.26^e^
	W2	2.07 ± 0.01^b^	77.63 ± 0.20^g^	3.68 ± 0.08e	12.58 ± 0.09^b^	13.11 ± 0.10^c^	73.67 ± 0.28^h^
	W3	1.65 ± 0.13^fgh^	81.10 ± 0.22^e^	3.22 ± 0.09f	11.89 ± 0.08^d^	12.32 ± 0.10^de^	74.86 ± 0.31^g^
	W4	1.49 ± 0.00^hi^	75.75 ± 0.21^h^	3.49 ± 0.07e	12.55 ± 0.11^b^	13.02 ± 0.12^c^	74.45 ± 0.26^gh^
	W5	1.62 ± 0.01^gh^	79.44 ± 0.58^def^	3.24 ± 0.15f	12.81 ± 0.17^b^	13.21 ± 0.20^c^	75.80 ± 0.50^f^
***mean wheat***		***1.77 ± 0.25***	***79.87 ± 3.71***	***3.12 ± 0.67***	***11.81 ± 1.49***	***12.22 ± 1.60***	***75.32 ± 1.60***
triticale	T1	1.76 ± 0.03^efg^	80.95 ± 0.10^e^	2.61 ± 0.02^gh^	10.42 ± 0.03^e^	10.74 ± 0.04^gh^	75.94 ± 0.07^f^
	T2	1.99 ± 0.02^bc^	80.93 ± 0.06^e^	2.77 ± 0.03^g^	9.85 ± 0.07^f^	10.24 ± 0.07^h^	74.27 ± −0.26^gh^
	T3	1.80 ± 0.03^def^	86.32 ± 0.09^a^	1.51 ± 0.02^m^	8.04 ± 0.14^k^	8.18 ± 0.14^m^	79.32 ± 0.21^c^
***mean triticale***		***1.85 ± 0.12***	***82.73 ± 3.11***	***2.30 ± 0.69***	***9.44 ± 1.25***	***9.72 ± 1.36***	***76.51 ± 2.57***
sorghum	S1	1.59 ± 0.00^gh^	70.14 ± 0.14^k^	4.69 ± 0.07^c^	9.96 ± 0.07^ef^	11.01 ± 0.08^g^	64.81 ± 0.32^j^
	S2	1.38 ± 0.01^i^	72.03 ± 0.09^j^	5.93 ± 0.06^b^	12.45 ± 0.12^bc^	13.79 ± 0.14^b^	64.53 ± 0.11^j^
	S3	1.86 ± 0.05^cde^	68.15 ± 0.34^l^	6.36 ± 0.08^a^	9.75 ± 0.04^f^	11.64 ± 0.08^f^	56.90 ± 0.26^k^
	S4	1.72 ± 0.04^efg^	73.64 ± 0.27^i^	3.97 ± 0.08^d^	9.71 ± 0.06^fg^	10.49 ± 0.09^gh^	67.79 ± 0.26^i^
***mean sorghum***		***1.63 ± 0.19***	***70.99 ± 2.37***	***5.24 ± 1.10***	***10.47 ± 1.32***	***11.73 ± 1.45***	***63.51 ± 4.64***
**mean wholegrain flour**		**1.90 ± 0.21**	**77.94 ± 6.79**	**2.71 ± 1.22**	**10.90 ± 1.71**	**11.29 ± 1.73**	**76.15 ± 5.94**

a *gen* = genotype; L* = brightness coordinate; a* = redness coordinate; b* = yellowness coordinate; C* = chroma; *and h*_ab_ = hue angle. Different letters in each column indicate significant differences (Tukey’s test, *p* < 0.05). Bold values are the mean values (mean ± SD) of cereal crops.

For the bran fraction, the ash content ranged from 3.12% (R3) to 6.62% (W2) with an average of 4.29% ± 0.94 (Table 4). Wheat bran presented the highest ash content (mean 5.66%), followed by triticale bran (5.28%), pearl millet (4.0%), barley (3.73%), rye (3.61%), and sorghum (3.47%). The S3 sample was the darkest bran sample with a brightness value of 55.65, while the three barley samples (B1–B3) presented the highest L* values (79.68–81.47). The three sorghum samples with colored pericarps (S1–S3) were the reddish bran samples, while T1, S2, and wheat brans were the samples with the most pronounced yellow color. The average ash content of the husk fraction was 4.24% ± 0.58, where B3 (5.62%) was significantly different from all other husks (Table 4). Barley husks (average of 70.48) were lighter than oat husks (average of 63.48). Both oat and barley husks showed positive redness and yellowness values, indicating that these samples are more red and yellow colored.

**Table 4:** Ash Content and Colorimetric Analysis of Bran and Husk Samples^a^

cereal crop	gen	ash (%)	L*	a*	b*	chroma	*h*_ab_
rye	R1	3.84 ± 0.01^i^	72.16 ± 0.26^d^	3.52 ± 0.06^k^	10.31 ± 0.21^gh^	10.90 ± 0.22^j^	71.14 ± 0.07^f^
	R2	3.86 ± 0.08^i^	74.66 ± 0.16^c^	3.10 ± 0.04^l^	10.86 ± 0.26^g^	11.29 ± 0.261	73.98 ± 0.04^d^
	R3	3.12 ± 0.07^hi^	74.18 ± 0.35^c^	3.08 ± 0.06^l^	9.62 ± 0.26^i^	10.10 ± 0.27^k^	72.25 ± 0.11^e^
***mean rye***		***3.61 ± 0.43***	***73.67 ± 1.33***	***3.23 ± 0.25***	***10.27 ± 0.62***	***10.77 ± 0.61***	***72.46 ± 1.43***
barley	B1	3.67 ± 0.08^fghi^	80.38 ± 0.37^ab^	2.49 ± 0.04^m^	10.63 ± 0.20^g^	10.92 ± 0.20^j^	76.81 ± 0.07^b^
	B2	3.20 ± 0.11^hi^	81.47 ± 0.24^a^	2.29 ± 0.05^m^	9.67 ± 0.19^hi^	9.94 ± 0.19^k^	76.69 ± 0.03^b^
	B3	4.31 ± 0.01^cdefgh^	79.68 ± 0.09^b^	3.08 ± 0.01^l^	12.22 ± 0.09^f^	12.61 ± 0.09^i^	75.83 ± 0.08^c^
***mean barley***		***3.73 ± 0.56***	***80.51 ± 0.90***	***2.62 ± 0.41***	***10.84 ± 1.29***	***11.15 ± 1.35***	***76.44 ± 0.53***
pearl millet	M1	4.15 ± 0.08^cdefgh^	63.79 ± 0.12^ij^	2.28 ± 0.07^m^	13.48 ± 0.23^cde^	13.67 ± 0.24^gh^	80.41 ± 0.15^a^
	M2	3.85 ± 0.05^efghi^	61.57 ± 0.16^k^	2.46 ± 0.02^m^	14.83 ± 0.10^b^	15.03 ± 0.10^e^	80.58 ± 0.06^a^
***mean pearl millet***		***4.00 ± 0.22***	***62.68 ± 1.57***	***2.37 ± 0.13***	***14.16 ± 0.95***	***14.35 ± 0.96***	***80.50 ± 0.12***
wheat	W1	5.23 ± 0.03^abcde^	74.02 ± 1.24^c^	4.49 ± 0.06^j^	12.89 ± 0.18^e^	13.65 ± 0.19^h^	70.78 ± 0.06^f^
	W2	6.62 ± 0.09^a^	67.04 ± 0.13^f^	6.16 ± 0.11^fg^	14.50 ± 0.26^b^	15.75 ± 0.28^d^	66.99 ± 0.19^h^
	W3	6.31 ± 0.05^ab^	65.80 ± 0.07^gh^	7.00 ± 0.05^d^	15.60 ± 0.10^a^	17.10 ± 0.10^ab^	65.83 ± 0.23^j^
	W4	4.95 ± 0.05^bcdef^	64.11 ± 0.08^i^	6.50 ± 0.10^e^	14.80 ± 0.19^b^	16.16 ± 0.22^cd^	66.30 ± 0.06^ij^
	W5	5.20 ± 0.01^abcdef^	67.38 ± 0.33^f^	6.12 ± 0.09^g^	14.45 ± 0.31^b^	15.69 ± 0.31^de^	67.05 ± 0.24^h^
***mean wheat***		***5.66 ± 0.75***	***67.67 ± 3.77***	***6.05 ± 0.94***	***14.45 ± 0.98***	***15.67 ± 1.26***	***67.39 ± 1.96***
triticale	T1	5.03 ± 0.03^bcdef^	65.27 ± 0.1^h^	6.38 ± 0.04^ef^	15.58 ± 0.16^a^	16.83 ± 0.16^bc^	67.72 ± 0.11^g^
	T2	5.42 ± 0.07^abc^	66.76 ± 0.11^fg^	5.93 ± 0.03^gh^	13.80 ± 0.19^c^	15.02 ± 0.18^ef^	66.73 ± 0.22^hi^
	T3	5.40 ± 0.04^abcd^	67.36 ± 0.22^f^	5.82 ± 0.14^h^	13.10 ± 0.31^de^	14.34 ± 0.34^fg^	66.06 ± 0.02^j^
***mean triticale***		***5.28 ± 0.22***	***66.47 ± 0.22***	***6.04 ± 0.30***	***14.16 ± 1.28***	***15.40 ± 1.29***	***66.84 ± 0.83***
sorghum	S1	3.36 ± 0.05^hi^	56.88 ± 0.24^l^	8.77 ± 0.17^c^	13.13 ± 0.28^de^	15.79 ± 0.32^d^	56.25 ± 0.21^m^
	S2	3.40 ± 0.00^ghi^	62.95 ± 0.26^j^	9.59 ± 0.04^b^	14.72 ± 0.07^b^	17.58 ± 0.06^a^	56.91 ± 0.12^l^
	S3	3.87 ± 0.11^cdefghi^	55.65 ± 0.36^m^	11.14 ± 0.17^a^	13.54 ± 0.26^cd^	17.53 ± 0.31^a^	50.56 ± 0.10^n^
	S4	3.30 ± 0.05^hi^	69.77 ± 0.03^e^	5.21 ± 0.07^i^	9.87 ± 0.04^hi^	11.16 ± 0.00^j^	62.16 ± 0.40^k^
***mean sorghum***		***3.47 ± 0.24***	***61.31 ± 6.48***	***8.68 ± 2.51***	***12.82 ± 2.08***	***15.52 ± 3.02***	***56.47 ± 4.75***
**mean bran**		**4.29 ± 0.94**	**68.72 ± 7.23**	**4.83 ± 2.50**	**12.78 ± 1.83**	**13.81 ± 2.26**	**70.02 ± 8.46**
oat	O1	3.81 ± 0.06^b^	63.88 ± 0.04^d^	5.69 ± 0.37^b^	20.62 ± 0.84^a^	21.39 ± 0.90^a^	74.58 ± 0.45^c^
	O2	3.90 ± 0.02^b^	65.42 ± 0.11^c^	6.78 ± 0.09^a^	21.20 ± 0.28^a^	22.26 ± 0.28^a^	72.26 ± 0.18^e^
	O3	3.77 ± 0.04^b^	61.13 ± 0.22^e^	4.78 ± 0.18^c^	16.21 ± 0.16^c^	16.90 ± 0.20^c^	73.56 ± 0.44^d^
***media oat***		***3.83 ± 0.07***	***63.48 ± 2.17***	***5.75 ± 1.00***	***19.34 ± 2.73***	***20.18 ± 2.88***	***73.46 ± 1.16***
barley	B1	4.04 ± 0.13^b^	71.12 ± 0.13^a^	3.35 ± 0.08^e^	14.04 ± 0.25^d^	14.43 ± 0.25^d^	76.59 ± 0.21^b^
	B2	4.26 ± 0.38^b^	69.93 ± 0.16^b^	4.18 ± 0.08^d^	16.96 ± 0.11^c^	17.47 ± 0.09^c^	76.14 ± 0.33^b^
	B3	5.62 ± 0.17^a^	70.40 ± 0.50^b^	4.16 ± 0.07^d^	19.41 ± 0.46^b^	19.86 ± 0.46^b^	77.91 ± 0.29^a^
***mean barley***		***4.64 ± 0.86***	***70.48 ± 0.60***	***3.90 ± 0.48***	***16.80 ± 2.69***	***17.25 ± 2.75***	***76.88 ± 0.92***
**mean husk**		**4.24 ± 0.58**	**66.98 ± 4.95**	**4.82 ± 1.31**	**18.07 ± 1.79**	**18.72 ± 2.07**	**75.17 ± 2.42**

a *gen* = genotype; L* = brightness coordinate; a* = redness coordinate; b* = yellowness coordinate; C* = chroma; and *h*_ab_ = hue angle. Different letters in each column indicate significant differences (Tukey’s test, *p* < 0.05) among the same milling fraction. Bold values are the mean values (mean ± SD) of cereal crops.

As expected, ash content progressively increased from flours (1.04) to wholemeals (1.90), bran (4.29%), and husk (4.24%) fractions, following the decrease of endosperm proportion. Accordingly, the brightness decreased from flours (86.90), wholemeal (77.94), brans (68.72), and husks (66.98). Similarly, Aprodu and Banu^34^ also found higher ash contents in whole cereals (wheat, rye, triticale, barley, and oat) samples than in flours, and they also obtained proportionally decreasing brightness values, according to the increase of outer layers’ presence.

### 3.2 Total Phenolic Content and Antioxidant Capacity of Cereal Samples

**Figure 2:**
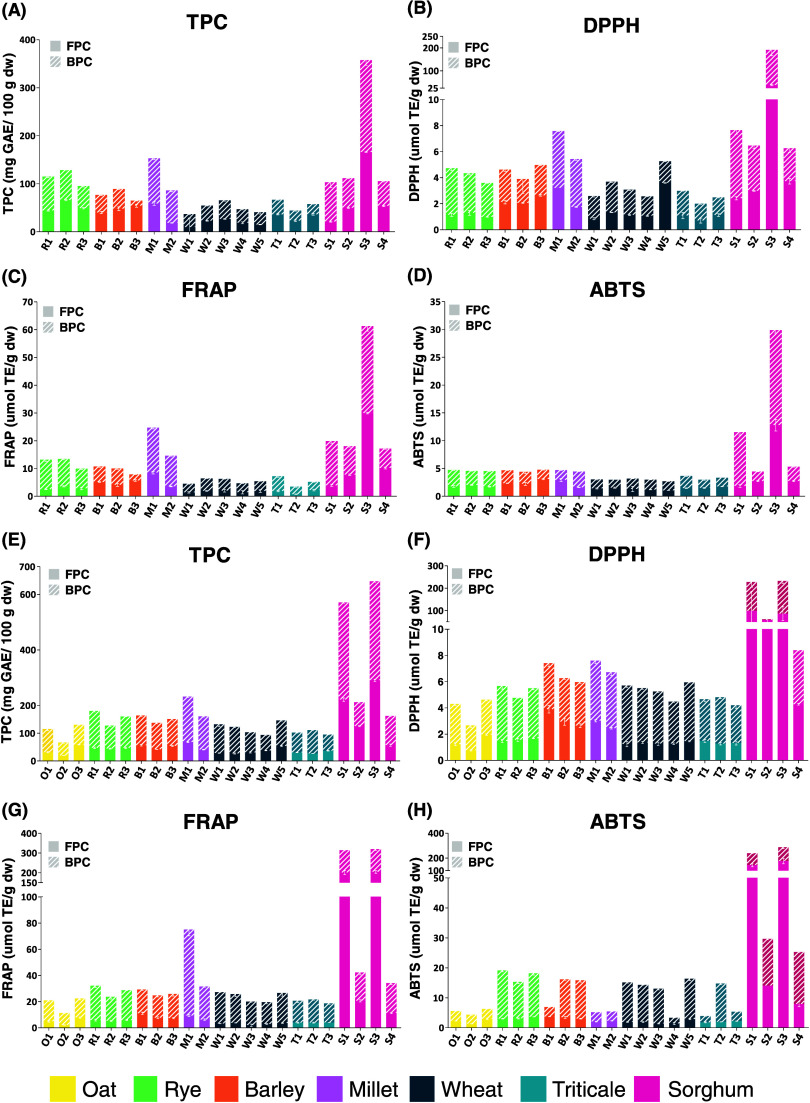
Total phenolic content and antioxidant capacity of flours (A–D) and wholegrain flours (E–H).

**Figure 3:**
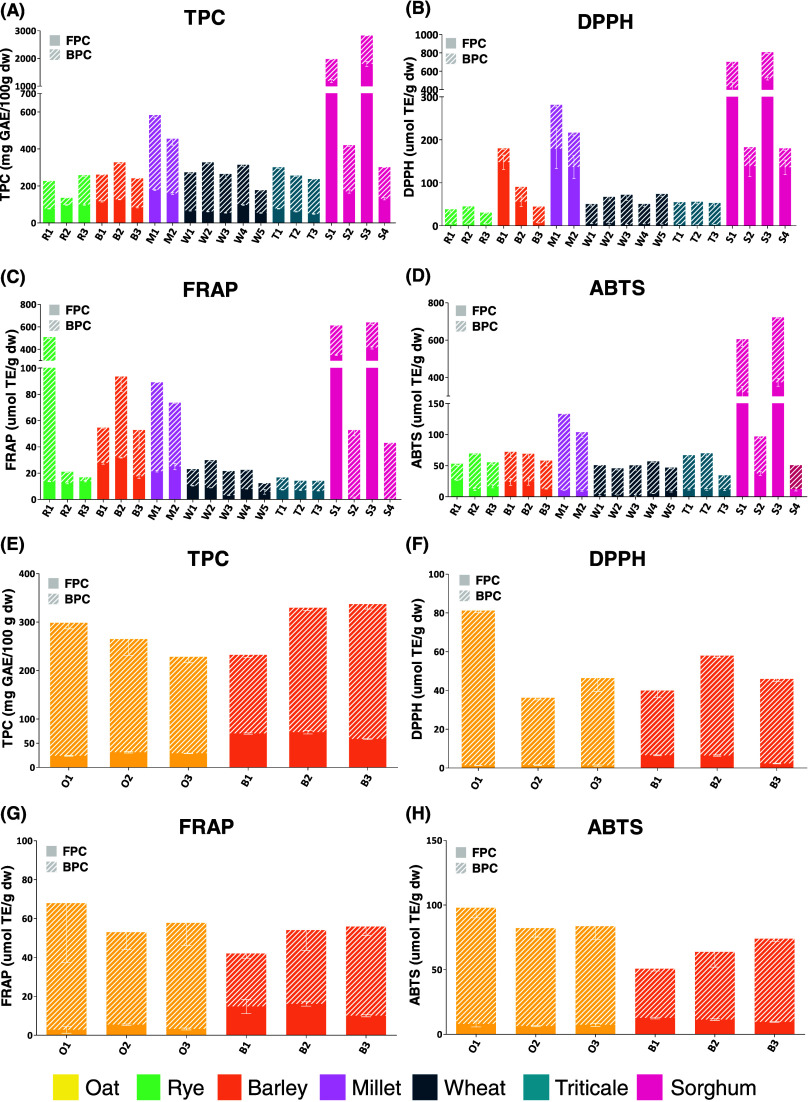
Total phenolic content and antioxidant capacity of bran (A–D) and husk samples (E–H).

The total phenolic contents of flour, wholemeal, bran, and husk fractions were estimated according to the classical method based on the extracts’ capacity to reduce the Folin-Ciocalteu reagent.^21^ The total phenolic content was calculated as the sum of the free and bound phenolic contents. A great variation between genotypes and cereal species among each milling fraction was found (Figures 2 and 3, please see the Supporting Information for complete data set table and statistical results, Tables S2–S5). Globally, bound extracts presented higher phenolic content and scavenging ability than the free extracts for all milling fractions, except for bran and wholemeal of tannin-rich sorghum (genotypes S1 and S3). In this case, for some of the antioxidant activity analyses, free extracts showed higher antioxidant activity than the bound ones. On average, bran presented the highest TPC and antioxidant capacity, followed by husk, wholegrain flour, and then flour samples, considering each milling fraction and the three methods based on the capacity of scavenging different radicals (DPPH and ABTS assays) and reducing the iron complex (FRAP assay).

#### 3.2.1 Flours

The free phenolic content of flours ranged from 15.02 mg (W5) to 165.98 mg GAE/100 g (S3) and averaged 43.70 ± 18.66 mg GAE/100 g, while the bound phenolic content ranged from 10.77 mg (B3) to 191.65 mg (S3) GAE/100 g, averaging 53.69 ± 30.38 mg GAE/100 g (Figure 2A–D and Table S2). The total phenolic content of flours averaged 97.39 ± 45.55 mg GAE/100 g and ranged from 40.81 (W5) to 357.62 (S3) mg GAE/100 g. The S3 flour showed the highest antioxidant capacity of the three methods performed here. The S3 genotype is a sorghum grain with brown pericarp, pigmented testa, and condensed tannins. Despite the S1 genotype having similar characteristics (light brown pericarp, pigmented testa, tannin-rich), S3 showed an outstanding antioxidant capacity about 10 times higher than the other flours (*p* < 0.05). However, other flour samples should be highlighted, such as the millet M1 and the other three sorghum genotypes, even those with no pigmented testa and tannin-free (S2, S4). Indeed, sorghum and millet grains, especially those with colored pericarp, have been highlighted to possess greater antioxidant capacity and total phenolic content when compared to wheat or rice, for instance.^8,35^

#### 3.2.2 Wholegrain Flours

The average free and bound phenolic contents of wholegrain flours were 61.17 ± 50.22 and 114.13 ± 54.91 mg of GAE/100 g, respectively (Figure 2E–G and Table S3). The total phenolic content averaged 175.30 ± 103.66 mg GAE/100 g and ranged from 66.61 (O2) to 647.00 (S3) mg GAE/100 g, with a great variation among the cereal species following the decrescent order: sorghum > pearl millet > rye > barley > wheat > oat > triticale. Tannin-rich sorghum (S1, S3) showed the greatest results. S3 showed the highest free phenolic content (290.49 mg GAE/100 g), and oat O2 had the lowest (19.48 mg GAE/100 g). For the bound phenolic content, S1 and S3 showed the highest amounts (349.84 and 356.51 mg GAE/100 g, respectively), whereas O2 (47.13 mg GAE/100 g), W4 (56.10 mg GAE/100 g), and T3 (58.36 mg GAE/100 g) presented the lowest contents. Apart from sorghum, other whole cereals can also be highlighted, such as pearl millet grains (M1, M2), rye R1 and R3, barley B1, and the sorghum genotypes tannin-free (S2, S4). These findings are consistent with those previously found, where colored sorghum presented a higher total phenolic content than pearl millet and white pericarp sorghum.^3^

Among the seven species here studied, triticale and oat samples presented the lowest total phenolic contents averaging 102.73 ± 8.04 and 103.98 ± 33.23 mg GAE/100 g, respectively, corroborating previous works performed in both species.^12,36^ Considering the remarkable difference found between the seven cereal species, environmental conditions, location, crop year, and even genetic factors can be pointed out as responsible for the variation in the phenolic content and composition of cereal grains.^12,37^

Tannin-rich sorghum genotypes (S1 and S3) presented the highest DPPH and ABTS scavenging results (*p* < 0.05) (Figure 2F–H). For FRAP assay, free extracts of S1, S3, and S2 expressed the highest scavenging ability, even S2 was statistically different from the others, although its value was 10 times lesser (20.58 ± 0.79 μmol TE/g) than S1 and S3 (∼200 μmol TE/g). The pearl millet M1 differed from the others, presenting the third highest FRAP result (75.09 ± 5.51 μmol TE/g), mainly due to its bound fraction (65.87 ± 4.82 μmol TE/g).

#### 3.2.3 Cereal Brans

Considering the averages of the total phenolic content, sorghum and pearl millet brans can be ranked as the highest amount of phenolic content (Figure 3A–D and Table S4). S3 had the highest amount (2,825.51 mg GAE/100 g), followed by S1 (1,975.94 mg GAE/100 g), M1 (583.32 mg GAE/100 g), M2 (455.29 mg GAE/100 g), and S2 (420.17 mg GAE/100 g), while R2 showed the lowest amount (135.37 mg GAE/100 g). On the other hand, barley, wheat, and triticale brans showed similar means, while rye bran had the lowest (206.97 mg GAE/100 g). The free phenolic content ranged from 48.63 (T3) to 1800.38 (S3) mg GAE/100 g, and the average was 219.97 ± 297.16 mg GAE/100 g. The range of bound phenolic content was 38.54–1024.63 mg GAE/100 g, where R2 showed the lowest content and S3, the highest. Excepting for S1 and S3 brans, bound fractions were the main factor responsible for the phenolic content in bran samples (Figure 3A). In summary, bran samples with the greatest scavenging potential in the three performed methods were sorghum S1, S2, and S3; pearl millet M1 and M2; and rye R1 and R2, although free extracts of barley B1 and B2 also showed a noticeable scavenging ability by the DPPH method.

#### 3.2.4 Cereal Husks

The total phenolic content average was 263.85 ± 35.17 mg of GAE/100 g for oat husk and 299.72 ± 58.39 mg of GAE/100 g for barley husk (Figure 3E–H and Table S5). B2 and B3 had the highest amounts of TPC (337.17 and 329.54 mg GAE/100 g, respectively), while O3 showed the lowest TPC (228.15 mg GAE/100 g). As expected, bound fractions represented most of the total phenolic content for both species (Figure 3E). In cereals, phenolic compounds are mainly found bound to hemicelluloses, such as arabinoxylans and *β*-glucans in barley and oats.^6^ The TPC of free extracts of barley husks (average of 67.13 mg of GAE/100 g) was significantly higher than that of oat free extracts (28.10 mg of GAE/100 g). Concerning the antioxidant activity assays, O3 (81.18 μmol of TE/g) showed the greatest activity by the DPPH assay, while O2 showed the lowest activity (36.20 μmol of TE/g). In FRAP analysis, no differences were found for total extracts in husks (free + bound). Sample O1 (97.86 μmol of TE/g) had the highest result, whereas B1 (50.70 μmol of TE/g) showed the smallest activity against the ABTS radical.

It is worth mentioning that oat and barley husks are byproducts of cereal milling, presenting a considerable volume in the industry since they can represent up to 30 percent of the entire grain. Besides phenolic compounds, these byproducts are fiber-rich materials, especially cellulose and hemicelluloses. Because of their valuable chemical composition, the development of nutraceutical ingredients and food packaging materials showing that biotechnological properties have been increasing in the last years,^38,39^ supporting the potential of bran and husk, the main byproducts of cereal milling.

In summary, the superior antioxidant activity and phenolic content of colorful sorghum grains were also significant in the present work. Similarly to our study, Nagy et al.^40^ also found that red and brown bran sorghum (with tannins) presented an antioxidant capacity and total phenolic content more than 10 times higher than white bran sorghum. Here, the tannin-rich sorghum genotypes (S1, S3) showed the highest phenolic content and antioxidant scavenging ability; however, the potential of the tannin-free red sorghum was also noticeable. Flavonoids are the main compounds related to the sorghum pericarp color variation, especially anthocyanins and condensed tannins subclasses.^4^ Li et al.^41^ found that the antioxidant capacity and total phenolic and flavonoid contents were similar among 11 genotypes of red sorghums. They also found that condensed tannins were significantly higher in soluble (free) fractions than in insoluble (bound) ones. Condensed tannins, also known as proanthocyanidins, are the main culprits for the antioxidant activity in sorghum. However, condensed tannins can complex sorghum proteins (kafirins), reducing their digestibility. In the last years, efforts have been made to increase sorghum protein digestibility through food clean-label technology, especially because of its interesting characteristics, such as bioactive potential, gluten-free, and neutral-flavor.^4,18,42^ Similarly, pearl millet grains were also remarkable. The interest in this minor crop has been increasing, especially due to the low water supply, resistance to drought, and tolerance to high temperatures.^2,4^ These advantageous physiological traits are relevant, and the potential of millet grains is noteworthy, considering the prospects of the rise of the human population and the climate changes in the earth for the next decades.

### 3.3 Metabolomics Revealing the Metabolite Diversity of Cereal Crops and Their Milling Fractions

To analyze the complex metabolomics data, different developed bioinformatic tools were used to maximize the data analysis comprehension and to explore as much as possible the high-throughput acquired data. The annotation step was carried out by combining GNPS spectral libraries and SIRIUS software (Figure 1) to have the highest possible number of annotated metabolites and thus have a global overview of the metabolic profile of the cereal samples.

Altogether, 102 metabolites were annotated in different confidence levels, 89 metabolites were putatively annotated (levels 2–3) and classified into different classes, including flavonoids, phenolic acids and derivatives, other polyphenols, organic acids, lipids and lipid-like molecules, amino acids, and their derivatives (Table 5). All of the data concerning the metabolites, such as *m/z*, mass error, and fragmentation pattern, are displayed in Table 5. In addition, the confirmation with analytical standards allowed us to identify (level 1) 13 phenolic compounds: *trans*-ferulic acid, *p*-coumaric acid, caffeic acid, 2,5-dihydroxybenzoic acid, sinapic acid, syringic acid, 4-hydroxybenzoic acid, myricetin, kaempferol, chlorogenic acid, (−)-epicatechin, apigenin, and vanillin.

**Table 5:** Annotated Compounds in the Cereal Samples by GNPS Libraries and SIRIUS^a^

no.	RT (min)	molecular formula	exp. [M – H]^−^*m/z*	theoretical [M – H]^−^*m*/*z*	error (ppm)	level	MS/MS fragments (% relative intensity)	proposed compound	GNPS	SIRIUS	VIP scores
Flavonoids											
1	4.08	C_15_H_12_O_7_	303.0511	303.0510	0.38	3	109.0275 (100), 175.0453 (51), 137.0239 (47), 83.0119 (32)	unknown flavonoid		x	2.404
2	5.05	C_30_H_26_O_12_	577.1390	577.1352	6.73	2	289.0704 (100), 125.0231 (65), 407.0748 (47), 161.0228 (24)	procyanidin B-type	x		
3	5.26	C_15_H_10_O_7_	301.0359	301.0354	1.74	3	NF	robinetin		x	2.667
4	5.29	C_15_H_14_O_6_	289.0722	289.0718	1.27	2	289.0722 (100), 109.0281 (30), 123.0438 (29)	luteoforol*		x	2.765
**5**	**5.92**	**C**_**15**_**H**_**14**_**O**_**6**_	**289.0722**	**289.0718**	**1.42**	**1**	179.0341 (100),135.0428 (36)	**(−)-epicatechin**		x	
6	6.00	C_22_H_18_O_11_	457.0810	457.0776	7.39	2	169.0127 (100)	epigallocatechin 3-gallate	x		
7	6.11	C_27_H_30_O_16_	609.1519	609.1461	9.43	2	327.0510 (100), 357.0603 (77), 309.0385 (26)	saponarin		x	2.064
8	6.15	C_46_H_44_O_18_	883.2406	883.2455	–5.53	3	433.0911 (100), 405.0659 (56), 287.0552 (16), 541.1036 (10)	procyanidin derivative		x	
9	6.38	C_14_H_10_O_6_	273.0402	273.0405	–1.05	2	121.0303 (51), 181.0496 (46), 110.9741 (43)	anomalin A		x	3.210
10	6.60	C_18_H_16_O_6_	327.0884	327.0874	3.01	2	327.0876 (100), 283.0965 (31), 163.0405 (22), 119.0483 (16)	betagarin		x	2.383
11	6.65	C_15_H_10_O_5_	269.0455	269.0455	–0.10	2	269.0455 (100), 117.0326 (19), 237.0753 (13)	prunetol*		x	2.852
12	6.78	C_27_H_30_O_15_	593.1563	593.1512	8.60	2	593.1563 (100), 401.1834 (22), 261.1335 (12), 269.0446 (9)	isovitexin 2″-*O*-glucoside		x	
13	6.90	C_15_H_14_O_6_	289.0719	289.0718	0.34	2	289.0719 (100), 125.0249 (71), 245.0820 (38), 179.0358 (18), 137.0249 (16), 109.0300 (11)	catechin*		x	
14	6.96	C_33_H_42_O_19_	741.2275	741.2248	3.71	2	271.0617 (100)	narirutin 4-glucoside		x	
15	7.13	C_22_H_22_O_13_	493.1007	493.0988	3.91	2	447.0911 (100)	isosakuranin	x	x	
16	7.18	C_22_H_18_O_10_	441.0854	441.0827	6.02	2	125.0242 (100), 169.0140 (34)	epicatechin 3-gallate	x	x	
17	7.26	C_22_H_26_O_11_	465.1420	465.1402	3.87	2	465.1412 (100), 303.0896 (55)	symplocoside		x	
18	7.34	C_16_H_12_O_5_	283.0613	283.0612	0.36	2	283.0613 (100), 253.0515 (37)	isoprunetin		x	2.415
19	7.42	C_15_H_12_O_7_	303.0518	303.0510	2.44	3	193.0500 (100), 177.0181 (19), 134.0362 (17)	unknown flavonoid		x	2.181
20	7.46	C_26_H_30_O_14_	565.1608	565.1563	8.00	2	271.0604 (100)	5,7,4′-trihydroxyflavanone 7-*O*-arabinosylglucoside	x	x	
21	7.57	C_15_H_12_O_7_	303.0515	303.0510	1.55	2	125.0213 (100), 285.0384 (12)	dihydroquercetin		x	3.718
22	7.89	C_27_H_30_O_14_	577.1592	577.1563	5.06	2	577.1592 (100), 271.0627 (10)	flavonoid dissacharide	x	x	
23	7.97	C_36_H_34_O_16_	721.1841	721.1774	9.28	2	721.1841 (100), 433.0958 (11)	5-*O*-β-d-glucosylluteoliflavan-(4 → 8)-eriodictyol		x	
24	8.40	C_16_H_14_O_6_	301.0722	301.0718	1.60	2	161.0241 (100)	sterubin		x	2.391^a^; 2.318^d^
**25**	**8.50**	**C**_**15**_**H**_**10**_**O**_**8**_	**317.0306**	**317.0303**	**0.90**	**1**	317.0306 (100),151.0038 (63),178.9978 (55),137.0240 (40)	**myricetin**	x		
26	9.05	C_21_H_20_O_9_	415.1057	415.1035	5.31	2	179.0337 (100), 253.0712 (14), 135.0432 (14)	daidzin*		x	
27	9.30	C_21_H_20_O_9_	415.1055	415.1035	4.92	2	415.1055 (100), 253.0720 (11), 161.0247 (7)	daidzin*		x	
28	9.61	C_15_H_12_O_6_	287.0563	287.0561	0.65	2	287.0563 (100), 135.0414 (78), 117.0355 (61)	dihydrokaempferol		x	2.676^a^; 2.379^b^
29	9.74	C_15_H_10_O_6_	285.0407	285.0405	1.00	2	285.0407 (100)	luteolin		x	2.342
30	9.92	C_16_H_14_O_6_	301.0722	301.0718	1.58	3	301.0722 (100), 243.0301 (42), 285.0407 (38), 239.0356 (37), 257.0439 (26)	unknown flavonoid		x	
31	10.08	C_22_H_22_O_9_	429.1213	429.1191	5.01	2	429.1213 (100), 193.0507 (51), 287.0944 (46), 317.1040 (21), 297.1356 (14), 285.0404 (14)	ononin		x	
32	10.54	C_19_H_16_O_8_	371.0787	371.0772	3.91	2	371.0787 (100), 247.0234 (16)	quercetin 3-isobutyrate		x	2.723
33	10.84	C_15_H_12_O_5_	271.0615	271.0612	1.12	3	NF	naringenin		x	2.952
34	**10.88**	**C_15_H_10_O_5_**	**269.0454**	**269.0455**	**–0.55**	**1**	**NF**	**apigenin**		x	2.329
35	10.98	C_16_H_14_O_6_	301.0717	301.0718	–0.21	3	301.0717 (100), 271.0593 (15)	unknown flavonoid		x	
36	11.06	C_16_H_12_O_6_	299.0563	299.0561	0.73	3	NF	unknown flavonoid		x	2.211
37	**11.08**	**C_15_H_10_O_6_**	**285.0403**	**285.0405**	**–0.57**	**1**	**NF**	**kaempferol**		x	
38	11.10	C_21_H_20_O_7_	383.1150	383.1136	3.58	2	329.2328 (100), 299.0533 (28)	gancaonin		x	2.497^a^; 2.451^d^
Phenolic Acids and Derivatives											
39	3.89	C_8_H_8_O_4_	167.0338	167.0350	–7.04	2	167.0338 (100), 123.044 (54)	dihydroxyphenylacetic acid	x		
40	4.32	C_7_H_6_O_4_	153.0181	153.0193	–7.98	2	109.0295 (100)	dihydroxybenzoic acid*		x	2.732
41	4.84	C_9_H_10_O_4_	181.0495	181.0506	–5.99	2	181.0495 (100), 163.0384 (11)	dihydrocaffeic acid		x	
42	5.07	C_15_H_18_O_9_	341.0886	341.0878	2.20	2	341.08886 (100), 179.0352 (55)	caffeoyl glucose		x	
**43**	**5.19**	**C**_**16**_**H**_**18**_**O**_**9**_	**353.0887**	**353.0878**	**2.40**	**1**	**191.0560 (100),137.0229 (13)**	**chlorogenic acid**		x	
**44**	**5.45**	**C**_**7**_**H**_**6**_**O**_**3**_	**137.0230**	**137.0244**	–10.35**	**1**	**93.0345 (100),109.0305 (21)**	4-hydrozybenzoic acid**		x	
**45**	**5.53**	**C**_**7**_**H**_**6**_**O**_**4**_	**153.0182**	**153.0193**	**–7.52**	**1**	**137.0224 (100)**	**2,5-dihydroxybenzoic acid**		x	
**46**	**5.88**	**C**_**9**_**H**_**8**_**O**_**4**_	**179.0341**	**179.0350**	**–5.05**	**1**	**179.0341 (100),135.0439 (40)**	**caffeic acid**	x		
47	5.98	C_16_H_18_O_8_	337.0939	337.0929	2.85	2	337.0939 (100), 93.0340 (60), 289.0734 (49), 233.1048 (43), 179.0341 (25), 131.0713 (23)	coumaroylquinic acid		x	
48	**6.00**	**C_9_H_10_O_5_**	**197.0446**	**197.0455**	**–4.81**	**1**	**179.0344 (100), 135.0424 (96)**	**syringic acid**		x	2.542
49	6.64	C_17_H_20_O_9_	367.1049	367.1035	3.94	2	191.0553 (100), 207.0664 (11)	feruloylquinic acid	x		
50	7.04	C_9_H_8_O_3_	163.0391	163.0401	–6.14	2	119.0500 (100)	coumaric acid*		x	
51	7.28	C_20_H_22_O_8_	389.1257	389.1242	3.77	3	163.0399 (100), 119.0501 (92), 193.0502 (32), 217.1080 (25)	hydroxycinnamic acid derivative		x	4.561
**52**	**7.29**	**C**_**9**_**H**_**8**_**O**_**3**_	**163.0389**	**163.0401**	**–7.02**	**1**	**119.0498 (100)**	***p*-coumaric acid**		x	2.395
**53**	**7.36**	**C**_**11**_**H**_**12**_**O**_**5**_	**223.0609**	**223.0612**	**–1.54**	**1**	**223.0609 (100),208.0371 (43),164.0467 (25),119.0509 (14),179.0706 (12)**	**sinapic acid**		x	
54	7.39	C_20_H_18_O_8_	385.0950	385.0929	5.37	2	341.1053 (100), 267.0644 (55), 282.0883 (39), 193.0502 (23), 223.0590 (19), 326.0783 (20)	diferulic acid*		x	
**55**	**7.40**	**C**_**10**_**H**_**10**_**O**_**4**_	**193.0501**	**193.0506**	**–2.77**	**1**	**193.0506 (100),178.0270 (61),134.0368 (48),149.0603 (34)**	***trans*-ferulic acid**	x		
56	7.56	C_11_H_10_O_5_	221.0450	221.0455	–2.55	2	221.0450 (100), 162.0319 (53), 177.0538 (30)	*p*-coumaroyl glycolic acid		x	
57	7.65	C_10_H_10_O_4_	193.0500	193.0506	–3.18	2	193.0500 (100), 134.0376 (96), 178.0270 (62), 149.0608 (41)	ferulic acid*	x		
58	7.81	C_20_H_18_O_8_	385.0950	385.0929	5.37	2	341.1045 (40), 297.1143 (17)	diferulic acid*	x		
59	8.41	C_20_H_18_O_8_	385.0946	385.0929	4.55	2	282.0906 (100), 173.0612 (96), 281.0836 (28), 123.0456 (26), 158.0452 (23), 341.1024 (18)	diferulic acid*		x	
60	8.71	C_20_H_18_O_8_	385.0947	385.0929	4.79	2	341.1022 (100)	diferulic acid*		x	
61	8.92	C_20_H_18_O_8_	385.0949	385.0929	5.10	3	NF	diferulic acid*		x	
62	5.34	C_12_H_14_O_6_	253.0716	253.0718	–0.64	2	253.0716 (100), 161.0250 (20), 153.0919 (10)	2-*O*-caffeoylglycerol		x	2.625
63	7.44	C_21_H_20_O_9_	415.1055	415.1035	5.03	2	193.0510 (100), 207.0302 (27), 134.0371 (13)	1,3-*O*-dicaffeoylglycerol		x	
64	6.00	C_25_H_31_N_3_O_6_	468.2173	468.2140	7.03	3	NF	*N*(1),*N*(8)-bis(caffeoyl)spermidine		x	3.217
65	9.45	C_17_H_15_NO_6_	328.0833	328.0827	1.86	2	328.0833 (100), 284.1873 (19)	avenanthramide 2f		x	
Other Polyphenols											
66	5.73	C_8_H_8_O_3_	151.0387	151.0401	–8.78	2	93.0337 (100), 136.0163 (47)	vanillin*		x	
67	6.57	C_11_H_14_O_5_	225.0762	225.0768	–2.87	2	123.0451 (100)	isovanilmandelic acid ethyl ester		x	2.185
**68**	**7.00**	**C**_**8**_**H**_**8**_**O**_**3**_	**151.0387**	**151.0401**	**–9.20**	**1**	**136.0157 (100)**	**vanillin**		x	
69	8.90	C_9_H_6_O_4_	177.0180	177.0180	0.00	3	NF	aesculetin		x	2.520
Lipid and Lipid-like Molecules											
70	2.63	C_6_H_10_O_5_	161.0443	161.0455	–7.74	2	161.0443 (100), 99.0434 (65)	meglutol	x		
71	8.47	C_12_H_20_O_5_	243.1235	243.1238	–1.31	3	225.1136 (100), 226.1205 (14)	fatty acid		x	
72	8.58	C_18_H_36_O_6_	347.2451	347.2439	3.42	3	NF	sativic acid		x	
73	8.90	C_12_H_20_O_5_	243.1235	243.1238	–1.31	3	NF	oxododecanedioic acid		x	
74	11.11	C_20_H_38_O_7_	389.2560	389.2545	3.84	2	329.2332 (100)	3-[(3,5-dihydroxydecanoyl)oxy]-5-hydroxydecanoic acid		x	2.050
75	12.38	C_18_H_34_O_5_	329.2343	329.2333	2.89	3	NF	fatty acid		x	
76	12.54	C_19_H_36_O_4_	[M + HCOO]^−^ 373.2608	373.2596	3.40	3	NF	nonadecandioic acid		x	2.899
Organic Acids											
77	1.04	C_6_H_12_O_7_	195.0506	195.0510	–2.41	2	195.0506 (100), 129.0179 (12)	gulonic acid		x	2.695
78	1.33	C_4_H_6_O_5_	133.0130	133.0142	–9.37	2	115.0032 (100), 71.0129 (13)	malic acid		x	
79	1.84	C_6_H_8_O_7_	191.0191	191.0197	–3.34	2	191.0191 (100), 111.0080 (70)	citric acid		x	
80	5.05	C_7_H_12_O_5_	175.0604	175.0612	–4.55	2	175.0604 (100), 115.0392 (65)	isopropylmalic acid		x	
Other Metabolites											
81	1.97	C_8_H_6_O_5_	181.0131	181.0142	–6.07	2	109.0291 (100), 111.0079 (22)	4-(furan-2-yl)-2,4-dioxobutanoic acid		x	2.788
82	2.26	C_9_H_12_N_2_O_6_	243.0621	243.0623	–0.48	2	85.0285 (100), 110.0245 (38), 129.0178 (23)	uridine		x	
83	3.05	C_26_H_40_O_20_	671.2106	671.2040	9.81	3	625.1963 (100), 179.0551 (15)	oligosaccharide		x	
84	4.89	C_12_H_12_O_7_	267.0511	267.0510	0.37	2	267.0511 (100), 221.0437 (73), 249.0420 (69), 222.0518 (26)	2-[(4-hydroxy-3,5-dimethoxyphenyl)methylidene]prop anedioic acid		x	2.150
85	4.71	C_11_H_12_N_2_O_2_	203.0821	203.0826	–2.50	2	203.0821 (100), 116.0509 (35), 159.0917 (19), 142.0652 (10)	tryptophan		x	
86	8.50	C_35_H_44_N_8_O_4_	639.3420	639.3413	1.06	3	385.0968 (94), 524.3113 (39), 474.2624 (36), 365.1011 (23)	amino acid derivative		x	2.026
87	8.63	C_23_H_27_NO_2_	348.1939	348.1969	–8.74	3	348.1939 (100), 164.0719 (25), 229.1581 (11), 181.0511 (11)	robustinin		x	2.562

a Compounds in bold were verified by the comparison to the reference standards. NF, not fragmented; exp., experimental; *isomers; ** mass error > 10 ppm, but it was verified by comparison to the analytical standard. Level of annotation: 1, compared to the authentic standards; 2, putatively annotation based on MS/MS spectral similarity with public libraries; and 3, putatively class annotation based on the MS/MS spectral similarity to other annotated compounds and/or classified by the CANOPUS tool on SIRIUS software. Letters in the VIP scores column mean: a, VIP in free extracts from cereal crops analysis; b, VIP in bound extracts from cereal crops analysis; c, VIP in free extracts from milling fractions analysis; and d, VIP in bound extracts from milling fractions analysis.

Flavonoids were the major class of annotated metabolites found as aglycone and glycosylated forms: flavanones (isosakuranin, naringenin, narirutin 4-glucoside, 5,7,4′-trihydroxyflavanone 7-*O*-arabinosylglucoside, 5-*O*-β-d-glucosylluteoliflavan-(4 → 8)-eriodictyol), flavones (apigenin, isovitexin 2″-*O*-glucoside), flavanols (epicatechin, catechin, epigallocatechin 3-gallate, epicatechin 3-gallate, procyanidin B, symplocoside), chalcones (dihydroxy-4-methoxychalcone-4′-*O*-neohesperid), flavonols (myricetin, kaempferol), and isoflavonoids (gancaonin, isoprunetin, daidzin, ononin).^16,17,43^

Hydroxycinnamic acids and their derivatives were the main phenolic acids found, despite the fact that hydroxybenzoic acids have also been detected: caffeic acid, *p*-coumaric acid and its isomer, sinapic acid, *trans*-ferulic acid and its isomer (*cis*-ferulic acid), dihydroxyphenylacetic acid, isomers of diferulic acids, 2,5-dihydroxybenzoic acid, dihydrocaffeic, 4-hydroxybenzoic acid, sinapic acid, syringic acid, and caffeoyl glucose. Hydroxycinnamic acids were also found conjugated with quinic acid: chlorogenic acid, feruloylquinic acid, and coumaroylquinic acid. In addition, avenanthramide 2f (*m*/*z* 328.08) was also annotated in the data set, which consists of the ferulic acid linked to an anthranilic acid. This ion presented the *m*/*z* 284 as the main fragment, indicating a typical loss of CO_2_ (−44 Da) and previously reported in oat grains.^12,44^ Furthermore, other phenolic acids derivatives were found: 1,3-*O*-dicaffeoylglycerol, 2-*O*-caffeoylglycerol, *p*-coumaroyl glycolic acid, and N(1),N(8)-bis(caffeoyl)spermidine.^43,45^

Five isomers of diferulic acid (*m*/*z* 385.09) were detected among the samples, with most of them showing *m*/*z* 341 as the main fragment, indicating a neutral loss of CO_2_ (−44 Da). These dehydrodimers of ferulic acids are mainly found in cereal grains cross-linked to polysaccharides in the cell wall. The ferulic acids can be linked to each other by ether or C–C bonds in different positions, composing different structures.^46^ Regarding the large number of possible structures due to the many linkage possibilities, it was not possible to determine the precise structure of the isomer. Diferulic acids were previously reported in cereal grains, including rye, sorghum, and wheat.^17,45,47^

**Figure 4:**
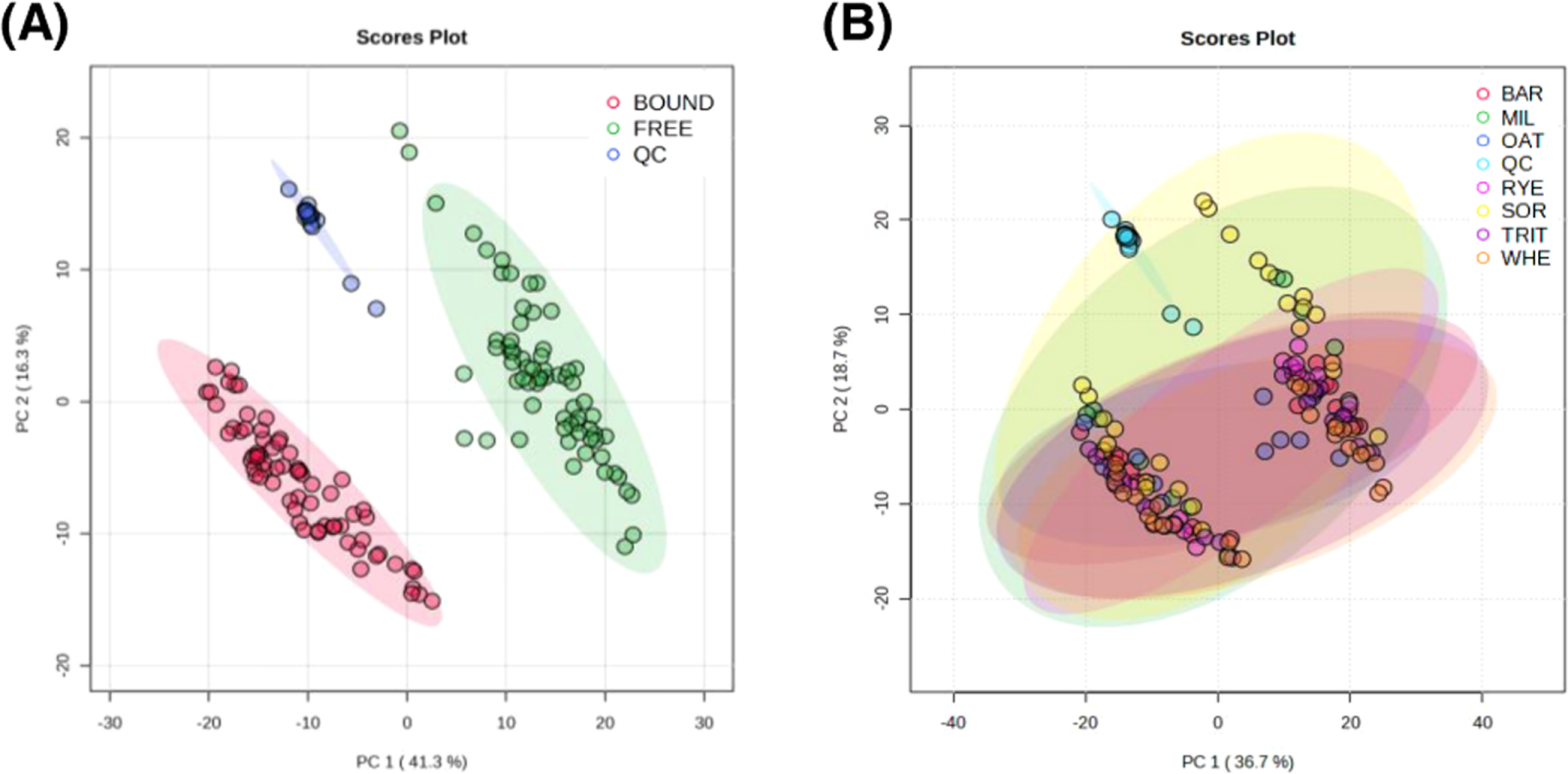
Principal component analysis (PCA) of the acquired metabolomics data. (A) PCA of samples grouped as free and bound extracts. (B) PCA of samples grouped as cereal crops.

Furthermore, the metabolomics data was submitted to multivariate data analyses to have an overview of the metabolite distribution in cereal crops and milling fractions. Unsupervised (principal component analysis, PCA; hierarchical clustering analysis, HCA) and supervised (partial least-squares-discriminant analysis, PLS-DA) methods were applied to analyze the data. First, a PCA (Figure 4) performed on all of the samples was applied to evaluate the quality of the acquired metabolomics data. It is possible to notice pooled QC samples clustered in the plot center, indicating that the data acquired were acceptable for further analysis. Additionally, PCA revealed a clear separation of free and bound extracts, indicating the first insights about the differences in the metabolite composition between both extracts. Furthermore, a detailed discussion about the chemometrics is provided in a further topic.

#### 3.3.1 Metabolite Composition Provided by the Feature-Based Molecular Networking

**Figure 5:**
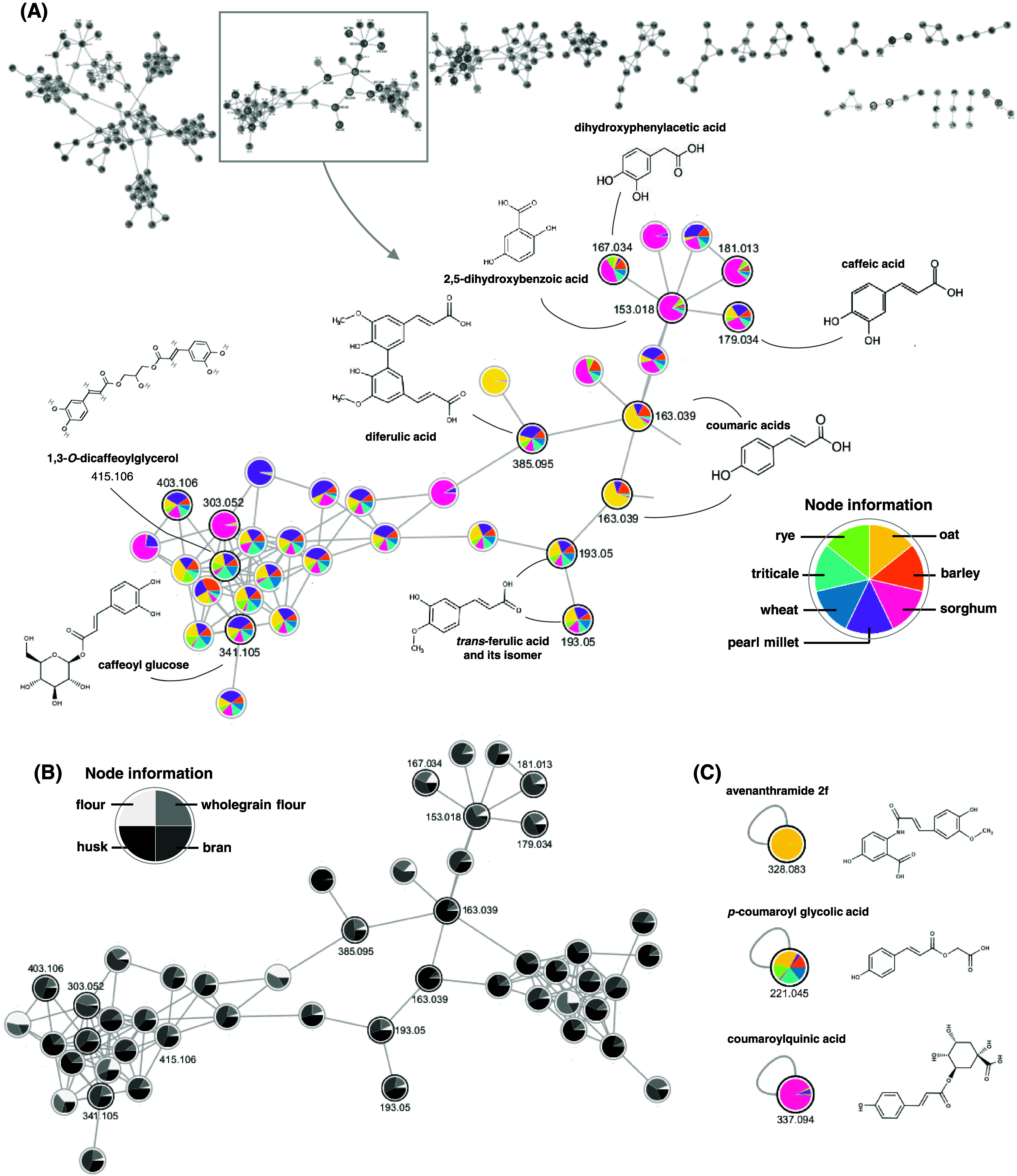
Feature-based molecular networking of cereal samples. (A) Major molecular networks provided by the FBMN analysis, and the second major family related to the phenolic acids. (B) Molecular networking of phenolic acids with an overview of the cereal milling fractions. (C) Other phenolic acid derivatives found in the cereal samples.

**Figure 6:**
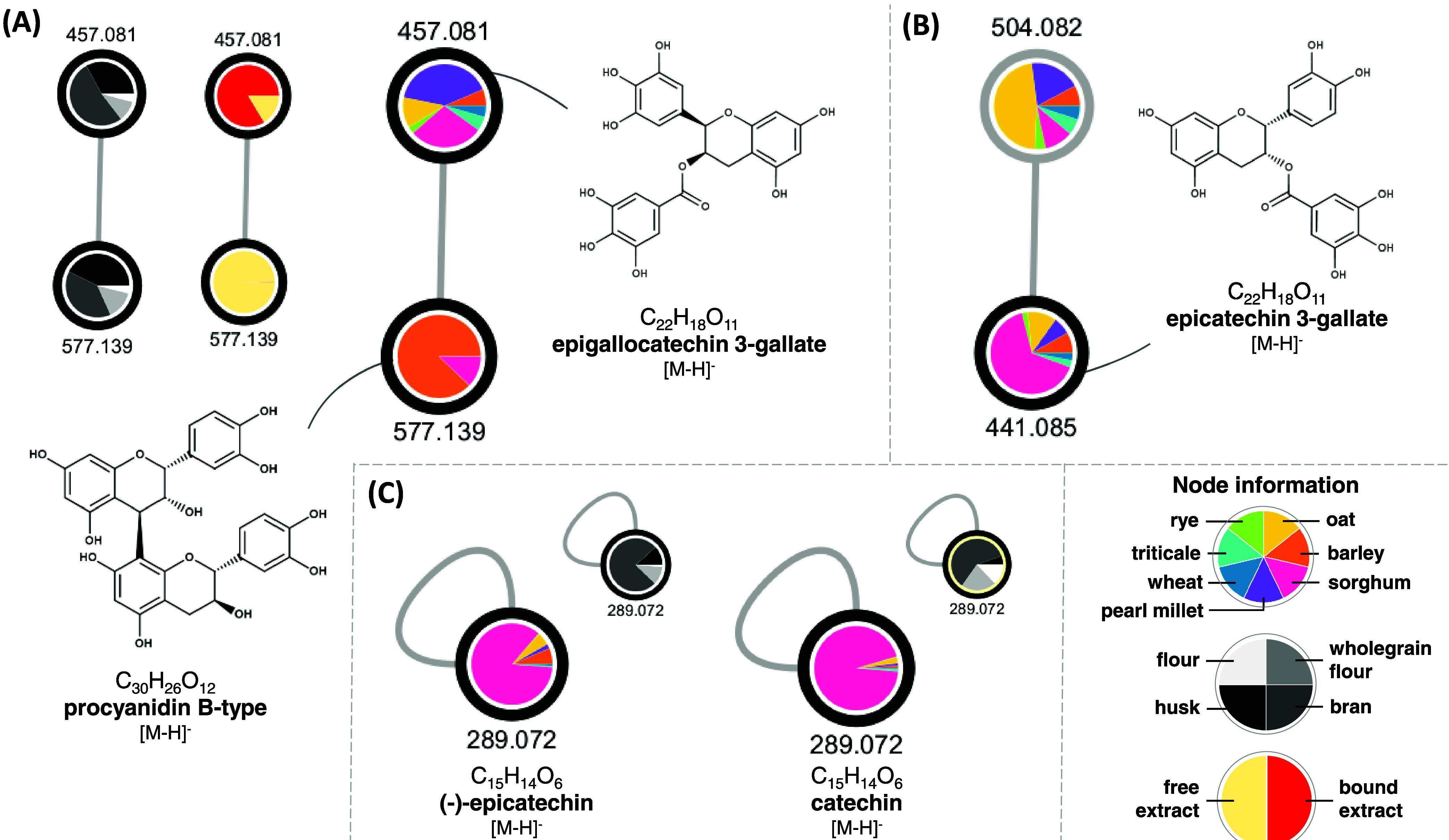
Feature-based molecular networking showing selected flavanols found in the cereal samples. (A) Nodes of the epigallocatechin 3-gallate and procyanidin B-type showing both flavanols in the cereal species, milling fractions, and extraction overview. (B) Node of epicatechin 3-gallate in the cereal species overview. (C) Nodes of (−)-epicatechin and catechin showing both flavanols in the cereal species and milling fraction overview. Node information shows the color meaning of each node.

The FBMN allowed for better visualization of the chemical profile of cereal crops, enabling a comprehensive overview of all of the variables explored in this study: cereal species, genotypes, milling fractions, and two different extracts. The FBMN analysis showed 1836 parent ions and presented eight major molecular networking with more than 4 nodes. Each node represents a metabolite (see node caption in Figures 5 and 6 for color meaning). Figure 5 highlights the second major cluster found by FBMN analysis, which showed 53 nodes and was related to the phenolic acids, including hydroxycinnamic and hydroxybenzoic acids and their derivatives. It was possible to highlight the following compounds in this molecular networking: *p*-coumaric acid and its isomer, caffeic acid, 2,5-dihydroxybenzoic acid, sinapic acid, *trans*-ferulic acid and its isomer (*cis*-ferulic acid), dihydroxyphenylacetic acid, caffeoyl glucose, 1,3-*O*-dicaffeoylglycerol, and one diferulic acid.

Most of the phenolic acids showed similar abundances among the seven cereal crops (Figure 5A). However, some of them could be highlighted because of their differences, such as the coumaric acids, which were mainly detected in oat samples due to the predominance of yellow color in the nodes. Also, diferulic acid was detected in all of the cereal crops but especially in millet grains, represented by the purple color in the node. Focusing on milling fractions, this compound showed more predominance in the bran fraction (Figure 5B, dark gray color). Another metabolite related to the hydroxycinnamic acids and exclusively found in oat samples was the avenanthramide 2f (*m*/*z* 328.08) (Figure 5C), which presented the *m*/*z* 284 as the main fragment, indicating a typical loss of CO_2_ (−44 Da) and being previously reported in oat grains.^12,44^ Regarding the milling fractions, phenolic acids showed predominance in bran and husk fractions, which are represented by the darkest colors in the molecular networking (Figure 5B).

A procyanidin B-type (*m*/*z* 577.14) was annotated by the GNPS library and presented as the main fragment ions *m*/*z* 289 and 125. Procyanidin was predominantly detected in barley and sorghum samples, which are highlighted by the orange and pink colors in the nodes (Figure 6A). Focusing on the milling fractions, this compound was particularly found in husk and bran fractions, highlighted as black and dark gray colors, respectively. Additionally, this flavonoid was predominantly detected in free extracts (light yellow color). Procyanidins are proanthocyanidins, composed of two (dimers), three (trimers), or more (epi)-catechin units, changing the hydroxylation and linkage between the isomers. The proanthocyanidins can also be composed of (epi)gallocatechin and (epi)afzelechin units, producing prodelphinidins and propelargonidins, respectively.^48^ These dimers were previously identified predominantly as free form in barley grains by Martínez et al.,^49^ representing something about 75% of the total free phenolics of their samples.

In sorghum, procyanidins are the main condensed tannins, exclusively found in the free form in colored pericarp grains.^4,43^ Xiong et al.^45^ found that procyanidin B1 and an isomer present more abundantly in the bran fraction of red and black sorghum than in the kernel. These results corroborate our findings, which were presented mainly as free forms in husk and bran fractions of sorghum and barley. The procyanidin B was linked to a node of *m*/*z* 457.08. This precursor presented *m*/*z* 169 as the main fragment ion and was annotated as epigallocatechin 3-gallate. This compound was detected mainly in pearl millet and sorghum samples (Figure 6A). Concerning the fractions, it was mostly detected in bound form (red color) and the bran and husk fractions (dark gray and black colors, respectively).

Another (epi)catechin derivative was annotated among the samples: epicatechin 3-gallate (*m*/*z* 441.08), which showed predominance in sorghum samples compared to the other cereal crops (Figure 6B) Moreover, the individual epicatechin and catechin (*m*/*z* 289.07) units were also annotated in the cereal samples, both showing more predominance in the bran fraction and sorghum samples (Figure 6C). As can be seen, sorghum was the cereal crop that presented more abundance of flavan-3-ols compounds, mainly in bran samples.

#### 3.3.2 Differences in the Metabolic Profile of Cereal Crops Provided by Chemometrics

**Figure 7:**
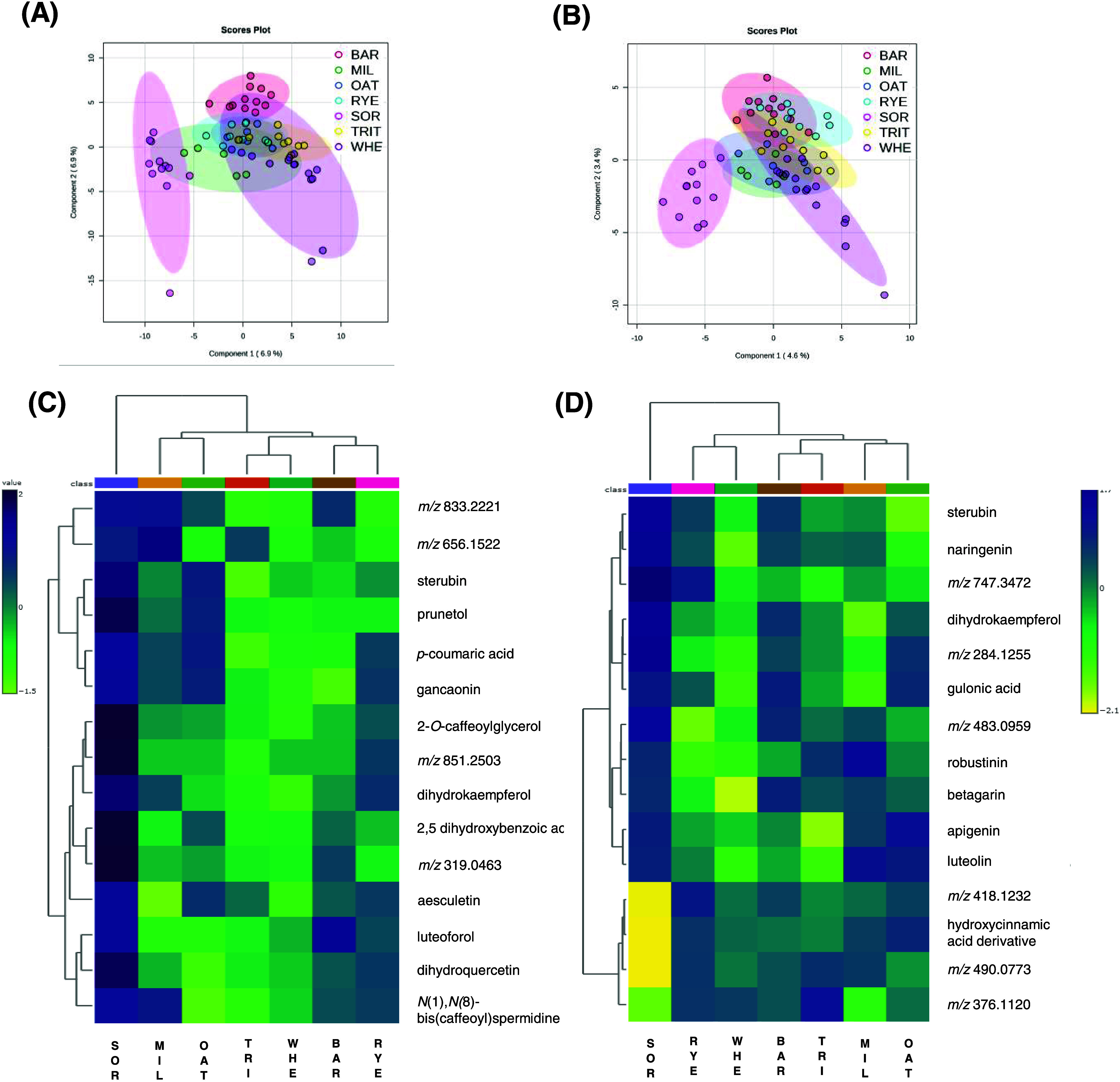
Multivariate data analysis of cereal crops. (A) Partial least-squares-discriminant analysis (PLS-DA) from free extracts of cereal samples. (B) PLS-DA from bound extracts of cereal samples. (C) Hierarchical cluster analysis (HCA) and heatmap visualization of the 15 most discriminant free metabolites among the cereal crops. (D) HCA and heatmap visualization of the 15 most discriminant bound metabolites among the cereal crops. BAR, barley; MIL, pearl millet; OAT, oat; RYE, rye; SOR, sorghum. TRIT, triticale; and WHE, wheat.

The PLS-DA was applied to discriminate the cereal crops and to extract the variable importance in the projection (VIP values), which represents the most discriminant metabolites among samples. Figure 7A displays the PLS-DA from free extracts that present as cross-validation parameters *R*^2^ = 0.201, *Q*^2^ = 0.027, whereas Figure 7B shows the model related to bound extracts, which presented *R*^2^ = 0.006 and *Q*^2^ = −0.038 for components 1 and 2. Both models were performed using 5-fold cross-validation due to the number of samples. In both analyses, sorghum samples were completely separated from the other cereals in free and bound extracts, indicating distinct metabolite composition. However, all of the other samples were clustered, and the model was not able to separate them, which can be related to the low parameters obtained in the cross-validation. Since that, a comparison grouping samples as sorghum samples and no-sorghum samples were made, and the cross-validation parameters from the PLS-DA model were *R*^2^= 0.910, *Q*^2^ = 0.692, and accuracy = 0.987 for free extracts; *R*^2^ = 0.982, *Q*^2^ = 0.477, and accuracy = 0.914 for bound extracts (Supporting Figure S1).

The 15 metabolites most important to differentiate the samples were extracted from the PLS-DA model and selected to be presented through the HCA and heatmap visualization. The HCA was built with Euclidean distance, grouping samples by cereal crops. Moreover, all of the metabolites presented VIP scores higher than 2.0 (Table 5). From the HCA analysis of free extracts (Figure 7C), three main clusters were generated, totally separating the sorghum samples from the others, corroborating our previous findings discussed above where sorghum samples showed a differential qualitative and quantitative phytochemical composition. The second cluster was divided into 2 clusters, in which pearl millet and oat samples were grouped and separated from triticale, wheat, barley, and rye samples. Interestingly, these four cereals share the same botanical tribe (Triticieae), differing from the other crops.

Flavonoids were the major metabolites responsible for the discrimination of cereal crops. Sterubin, luteoforol, gancaonin, dihydroquercetin, prunetol, and dihydrokaempferol were designated as VIPs for free extracts and presented the highest abundance in sorghum samples. The phenolic acids *p*-coumaric acid and 2,5-dihydroxybenzoic acid also contributed, presenting the greatest abundance in sorghum, rye, and pearl millet. In addition, 2-*O*-caffeoylglycerol and N(1),N(8)-bis(caffeoyl)spermidine were also noticed as being responsible for the separation of the cereal species, in which sorghum and pearl millet predominantly presented these compounds. Both compounds were previously reported in cereals, especially in sorghum, millets, and wheat.^17,43,50^

Similarly to the free extracts, the HCA of bound extracts (Figure 7D) showed flavonoids (sterubin, naringenin, dihydrokaempferol, betagarin, luteolin, and apigenin) as the main class contributing to the cereal crops’ discrimination. In addition, gulonic acid (organic acid) and carbazole (robustinin) were annotated. Also, the *m*/*z* 389.12 was annotated as a hydroxycinnamic acid derivative, since it presented the *m*/*z* 163, 119, 193, and 217 as major MS/MS fragments.

**Figure 8:**
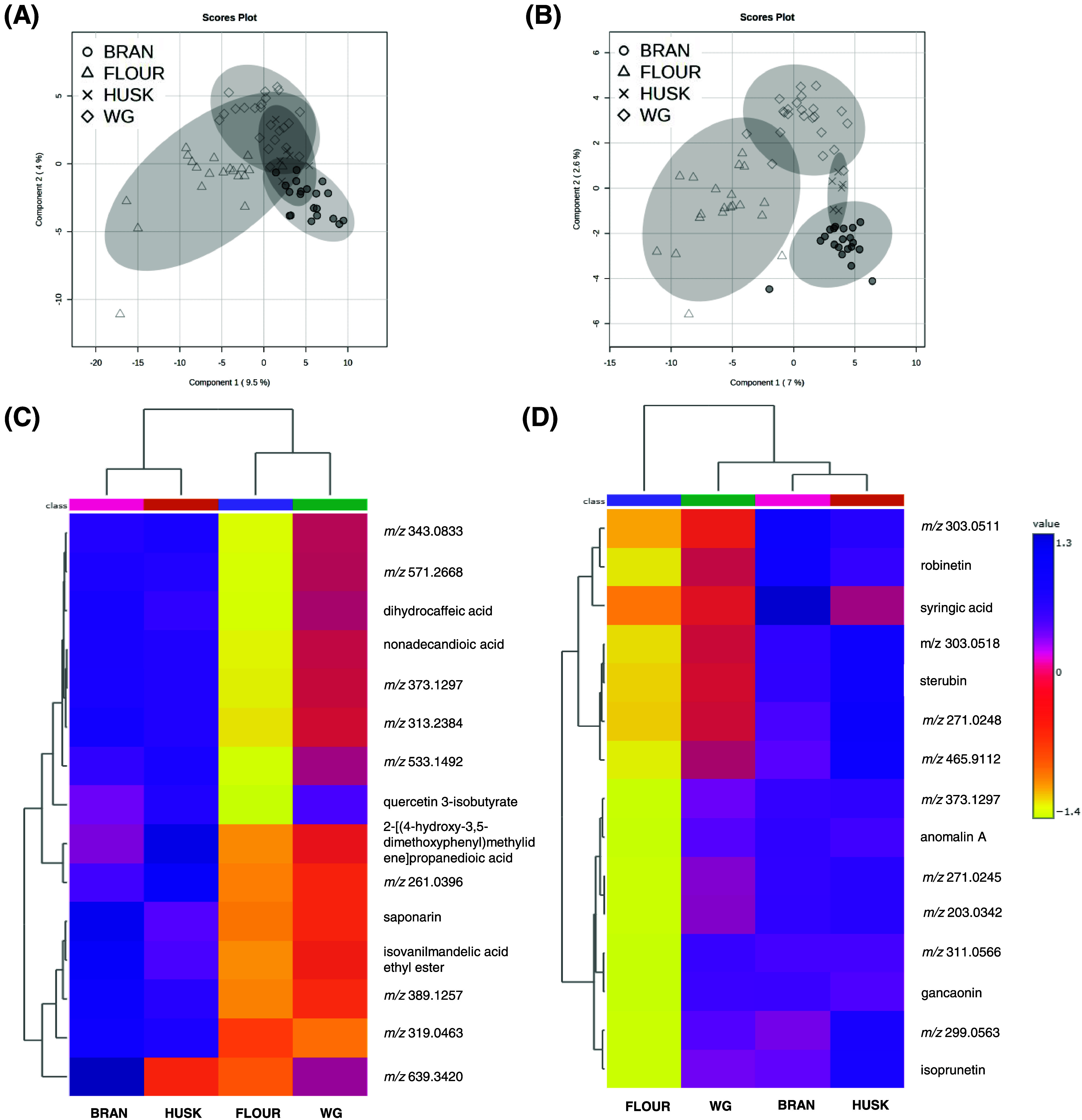
Multivariate data analysis of milling fractions. (A) Partial least-squares-discriminant analysis (PLS-DA) of metabolomics data from free extracts. (B) PLS-DA from bound extracts. (C) Hierarchical cluster analysis (HCA) and heatmap visualization of the 15 most discriminant free metabolites among the milling fractions. (D) HCA and heatmap visualization of the 15 most discriminant bound metabolites among the milling fractions.

Figure 8 displays the results of the supervised analyses concerning the comparison of the samples by milling fractions groups. The PLS-DA from free extracts (Figure 8A) revealed samples presented similarities in the metabolite composition, since there was not a full separation of the milling fractions, despite the fact that brans and flours were distinguishable from each other. The cross-validation parameters of this model were *R*^2^ = 0.970 and *Q*^2^ = 0.342. On the other hand, the PLS-DA of bound extracts (Figure 8B) revealed a clear separation of flours, wholegrain flours, bran, and husk fractions, suggesting that the four milling fractions have distinguished metabolomic profiles. The R2 value for this model was 0.981, and *Q*^2^ = 0.388. The low values for the acquired cross-validation parameters from the PLS-DA models, especially *Q*^2^ values, may indicate that the models are not predictive.

Figure 8C,D displays the HCA and heatmap with the abundance of the 15 most important metabolites in distinguishing the free and bound extracts of the milling fractions, respectively. Flours showed the lowest abundance of these metabolites in both models, whereas bran and husk fractions presented the highest. Phenolic acids, flavonoids, fatty acyls, and other polyphenols were the main culprits for the differences between free extracts of bran and husk from the flour and wholegrain flour. Flavonoids (isoprunetin, ganconin, sterubin, anomalin A, and robinetin) were the metabolites with higher abundance in the bran and husk. Syringic acid presented the highest abundance in the bran fraction than in the others. Nonetheless, we could not provide the putative annotation of some metabolites indicated as VIP; thus, for these metabolites, we provided their acquired [M – H]^−^*m*/*z*, VIP scores, and the complete MS data, such as fragment pattern and molecular formula (Supporting Table S3).

### 3.4 Correlations Between Physicochemical Analysis, Phenolic Content, Antioxidant Activity, and Metabolite Composition

Strong positive correlations were found between TPC and the three antioxidant assays (DPPH, *r* = 0.961; FRAP, *r* = 0.841; ABTS, *r* = 0.974) (Table 6), indicating that samples with the highest phenolic content also showed the greatest antioxidant capacity. The antioxidant assays were also strongly positively correlated to each other: FRAP and ABTS (*r* = 0.876), DPPH and FRAP (*r* = 0.822), and DPPH and ABTS (*r* = 0.957). Another strong positive correlation was found between the metabolite composition (calculated by the total relative abundance of the metabolites) and the phenolic content (*r* = 0.806) and DPPH (*r* = 0.823), despite significant correlations between the metabolomics analysis and ABTS (*r* = 0.796) and FRAP (*r* = 0.655) assays also been found. These results indicate that samples with high antioxidant capacity also showed the highest relative abundance of metabolites. Significant negative correlations were found between brightness and ash content (*r* = −0.658) and brightness and metabolomics (*r* = −0.793), meaning that the whitest (refined) cereal samples showed the lowest ash content and relative abundance of metabolites. These results were verified by the comparison between flours and other milling fractions in the ash content and by the PLS-DA model in which flour samples presented the lowest abundance of the VIP metabolites.

**Table 6:** Correlation Matrix Between Ash Content, Colorimetry, Total Phenolic Content, Antioxidant Capacity, and Metabolomic Analysis of Cereal Samples (Pearson Correlation Coefficients, *r* value, *p* < 0.05)^a^

	TPC	DPPH	FRAP	ABTS	Ash	L*	a*	b*	LC–MS
TPC	1								
DPPH	**0.961**	1							
FRAP	**0.841**	**0.822**	1						
ABTS	**0.974**	**0.957**	**0.876**	1					
Ash	0.259	0.220	0.161	0.252	1				
L*	–0.541	–0.545	–0.450	–0.543	**–0.658**	1			
a*	**0.640**	**0.629**	0.511	**0.647**	**0.669**	**–0.815**	1		1
b*	0.224	0.197	0.094	0.222	0.643	**–0.749**	**0.632**	1	
LC–MS	**0.806**	**0.823**	**0.655**	**0.796**	0.459	**–0.793**	**0.803**	0.482	1

a ASH, ash content; TPC, total phenolic content estimation; FRAP, FRAP analysis; DPPH, DPPH analysis; ABTS, ABTS analysis; L*, brightness; a*, redness coordinate; b*, yellowness coordinate; TPC, total phenolic content; and LC–MS, metabolomic analysis. Bold values mean the most significant correlations.

**Figure 9:**
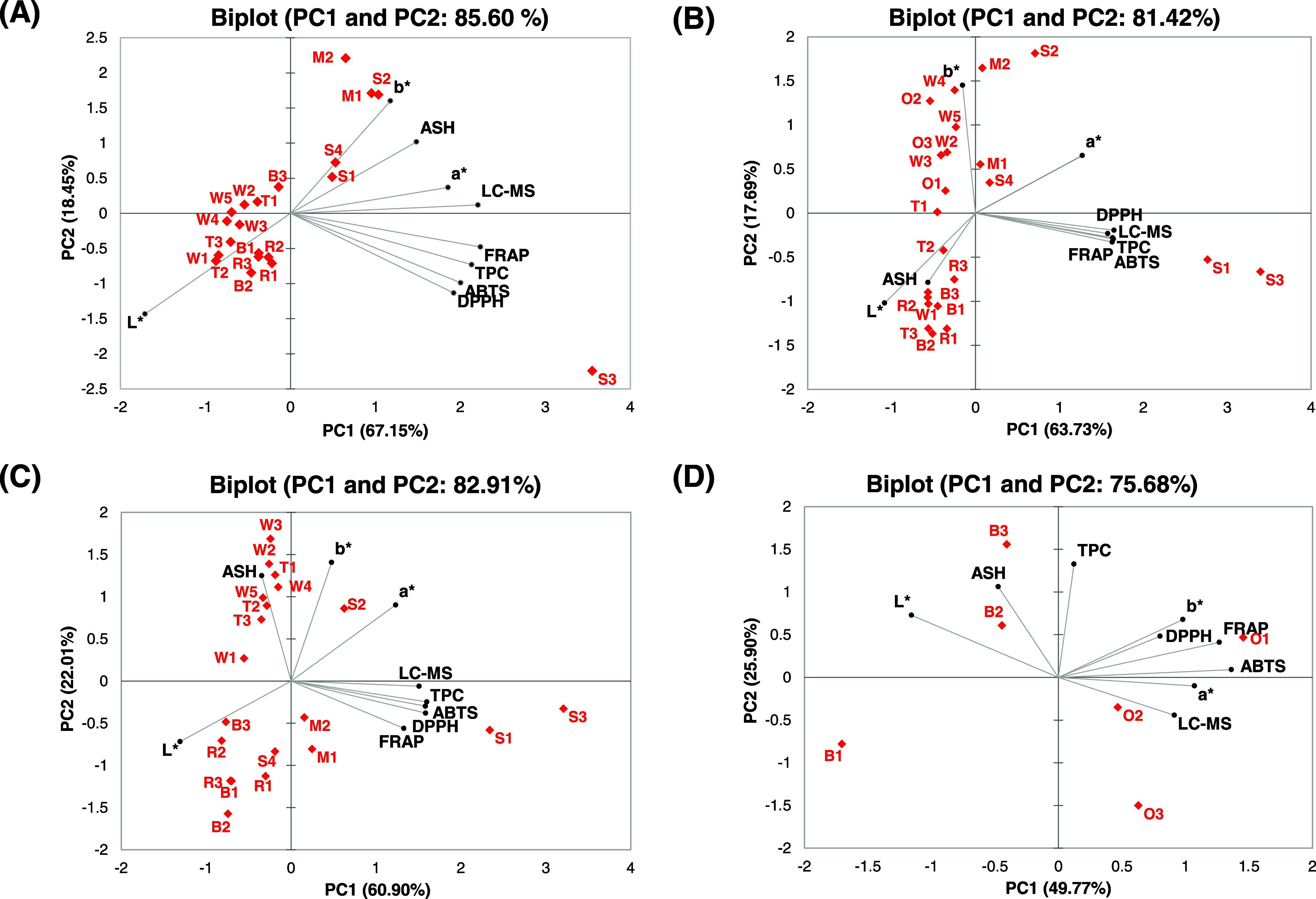
Principal component analyses (PCA) biplot of physicochemical, antioxidant activity, phenolic content, and metabolomics analyses of flours (A), wholegrain flours (B), brans (C), and husk fractions (D). Dot: dependent variables (results); diamond: samples. ASH, ash content; L*, brightness; a*, redness coordinate; b*, yellowness coordinate; TPC, total phenolic content; and LC–MS, total relative abundance of free and bound extracts provided by the metabolomics analysis.

Biplot PCA was also built to explore and correlate the antioxidant capacity, total phenolic content estimation, ash content, colorimetry, and metabolomics analyses (Figure 9). Figure 9A shows the PCA biplot from flours, in which PC1 explained 67% of the variance and was related to the three antioxidant assays, metabolomics analysis, ash content, redness, and yellowness coordinates. From this model, PC1 separates sorghum and pearl millet flours from the other flours. Similarly, whole sorghum and pearl millet flours were separated from the other wholegrain flours (Figure 9B). The sum of PC1 and PC2 was 81%, and PC1 (64%) was associated with antioxidant analyses, metabolomics analysis, total phenolic content, and redness coordinate. PC2 was responsible for 18% of the variance and separation of whole barley, triticale, and rye flours from whole oats and wheat flours except for W1. The PCA biplot from bran samples showed PC1 (61%) associated with antioxidant analyses, metabolomics analysis, redness, and yellowness coordinates (Figure 9C). From this analysis, colored pericarp sorghum (S1–S3) and pearl millet brans were separated by PC1 from the other bran samples. PC2 (22%) separates barley and rye brans from triticale and wheat brans. Figure 9D displays the PCA biplot from the husk samples, where the sum of PC1 and PC2 was 76%. Oat husks were clearly separated from the barley husks by PC1 (50%), in which this component was associated with metabolomics analysis, total phenolic content, antioxidant capacity, redness, and yellowness coordinates.

This work seems to be the first in characterizing and comparing through high-resolution metabolomic approaches major and minor cereal crops, concerning both different milling fractions and the number and diversity of species, which differ in the botanical taxonomy. Analyzing all of these different samples under the same conditions (*e.g.*, using the same metabolite extraction procedure, metabolomics workflow, and antioxidant assays) allows us a more confident comparison among samples, since they were treated equally. Additionally, this work contributes to providing metabolomics data of pearl millet and, especially, of triticale grains, which still shows a lack of information when analyzed by metabolomics approaches.

The influence of tannins in the antioxidant capacity, total phenolic content, and distinct metabolite profiling was noticeable, drawing attention to outstanding tannin-rich sorghum genotypes. Sorghum and millet appeared as distinct crops, and even those with no pigmented testa and tannin-free also exhibited great results. The milling processing also affects the qualitative and quantitative composition of the cereal fractions, in addition to the metabolite composition. Refined flours showed the lowest antioxidant capacity, phenolic content, ash content, and relative abundance of specialized metabolites. Remarkably, byproducts of cereal milling (husk and bran) showed the greatest antioxidant potential, phenolic content, and relative abundance of metabolites compared to the flours and wholegrain flours. In this way, this work shows the bioactive potential of byproducts and adds evidence to further studies.

## Supporting Information Available

Moisture content of cereal samples (Supporting Table S1); total phenolic content and antioxidant capacity of flour samples (Supporting Table S2); total phenolic content and antioxidant capacity of wholegrain flours (Supporting Table S3); total phenolic content and antioxidant capacity of bran samples (Supporting Table S4); total phenolic content and antioxidant capacity of husk samples (Supporting Table S5); discrimination of annotated metabolites per sample (Supporting Table S6); list of the unknown metabolites highlighted by the PLS-DA models (Supporting Table S7); PLS-DA models and cross-validation parameters from the comparison between sorghum samples and no-sorghum samples (Supporting Figure S1) (PDF)

## Abbreviations and Nomenclature

AACCI: American Association of Cereal Chemists International
ABTS: 2,2′-azino-bis(3-ethylbeenzothiazoline-6-sulfonic acid) diammonium salt
ANOVA: analysis of variance
BPC: bound compounds
CE: catechin equivalents
DW: dry weight
FPC: free compounds
FRAP: ferric-reducing antioxidant power
GAE: gallic acid equivalents
HCA: hierarchical cluster analysis
PCA: principal component analysis
PLS-DA: partial least-squares-discriminant analysis
QC: quality control
TPC: total phenolic content
Trolox: 6-hydroxy 2,5,7,8-tetramethylchroman-2-carboxylic acid
TE: trolox equivalents
UHLPC–HRMS: ultrahigh performance liquid chromatography coupled to high-resolution mass spectrometry
VIP: variable importance for the projection
