# Supplementary material for: Overview of the Metabolite Composition and Antioxidant Capacity of Seven Major and Minor Cereal Crops and Their Milling Fractions
Source: J Agric Food Chem. 2024 May 28;72(34):19197–218. doi: 10.1021/acs.jafc.4c01312 (PMC11363145; doi:10.1021/acs.jafc.4c01312)

An overview of the metabolite composition and antioxidant capacity of seven major and minor cereal crops and their milling fractions

Luciana Ribeiro da Silva Lima[ab], Millena C. Barros Santos[c], Paulo Wender P. Gomes[d], Álvaro Fernández-Ochoa[e], Mariana Simões Larraz Ferreira[ab]*

[a]Laboratory of Bioactives, Food and Nutrition Graduate Program (PPGAN), Federal University of the State of Rio de Janeiro (UNIRIO), Rio de Janeiro, 22290-240, Brazil;

[b]Center of Innovation in Mass Spectrometry, Laboratory of Protein Biochemistry, UNIRIO, Rio de Janeiro, 22290-240, Brazil;

[c]Bordeaux Metabolome-MetaboHUB, INRAE Bordeaux Nouvelle-Aquitaine, Villenave d'Ornon, UMR1332 BFP, France;

[d]Collaborative Mass Spectrometry Innovation Center, Skaggs School of Pharmacy & Pharmaceutical Sciences, University of California San Diego, 9500 Gilman Drive, La Jolla, San Diego, CA, 92093-0751, United States;

[e] Department of Analytical Chemistry, Faculty of Sciences, University of Granada, Granada, 18071, Spain.

*E-mail: mariana.ferreira@unirio.br; Phone: +55 (21) 2542-7269

Supplementary Table S1. Moisture content of cereal samples.

cereal crop	code	flour (%)	wholegrain flour (%)	bran (%)	husk (%)
oat	O1	N/A	11.83 ± 0.23	N/A	10.93 ± 0.09
	O2	N/A	11.49 ± 0.05	N/A	10.44 ± 0.19
	O3	N/A	11.21 ± 0.48	N/A	10.42 ± 0.08
rye	R1	12.03 ± 0.12	12.32 ± 0.16	11.02 ± 0.24	N/A
	R2	12.65 ± 0.29	12.41 ± 0.35	11.43 ± 0.19	N/A
	R3	12.32 ± 0.09	12.83 ± 0.20	11.63 ± 0.36	N/A
barley	B1	12.77 ± 0.16	12.64 ± 0.08	11.71 ± 0.10	11.95 ± 0.29
	B2	12.86 ± 0.37	12.54 ± 0.13	11.77 ± 0.36	11.20 ± 0.09
	B3	12.74 ± 0.04	12.51 ± 0.11	11.88 ± 0.09	10.86 ± 0.13
pearl millet	M1	13.39 ± 0.03	13.05 ± 0.14	12.54 ± 0.07	N/A
	M2	12.66 ± 0.16	11.67 ± 0.03	11.96 ± 0.11	N/A
soft wheat	W1	12.79 ± 0.14	12.57 ± 0.08	12.21 ± 0.21	N/A
	W2	12.51 ± 0.08	12.16 ± 0.09	12.07 ± 0.06	N/A
	W3	12.53 ± 0.13	12.36 ± 0.03	11.84 ± 0.04	N/A
	W4	12.42 ± 0.20	11.19 ± 0.18	10.81 ± 0.06	N/A
	W5	12.83 ± 0.12	11.87 ± 0.05	10.90 ± 0.03	N/A
triticale	T1	12.20 ± 0.03	12.10 ± 0.07	11.18 ± 0.10	N/A
	T2	11.20 ± 0.07	9.52 ± 0.07	10.44 ± 0.09	N/A
	T3	11.42 ± 0.06	13.56 ± 0.16	10.70 ± 0.14	N/A
sorghum	S1	12.66 ± 0.52	12.88 ± 0.10	11.32 ± 0.09	N/A
	S2	12.08 ± 0.07	13.11 ± 0.19	10.87 ± 0.12	N/A
	S3	12.61 ± 0.21	14.10 ± 0.12	11.11 ± 0.04	N/A
	S4	12.69 ± 0.14	13.47 ± 0.11	11.10 ± 0.05	N/A

Data presented are the mean values (mean ± SD, n=3); N/A, not applicable.

Supplementary Table S2. Total phenolic content and antioxidant capacity of flour samples.

flours		**TPC (mg GAE/100 g dw)**			**DPPH (µmol TE/g dw)**			**FRAP (µmol TE/g dw)**			**ABTS (µmol TE/g dw)**		
**cereal**	**gen**	**free**	**bound**	**total**	**free**	**bound**	**total**	**free**	**bound**	**total**	**free**	**bound**	**total**
rye	R1	44.29 ± 1.22ef	70.84 ± 2.39cd	115.14 ± 3.61cd	1.15 ± 0.12	3.58 ± 0.18b	4.73 ± 0.30b	2.50 ± 0.09hij	10.69 ± 0.68c	13.19 ± 0.77e	1.75 ± 0.16efgh	2.98 ± 0.09c	4.73 ± 0.25cd
	R2	66.11 ± 1.53b	62.40 ± 0.10de	128.51 ± 1.63c	1.30 ± 0.19	3.04 ± 0.12b	4.34 ± 0.31b	3.55 ± 0.20fg	9.90 ± 0.30c	13.45 ± 0.50e	2.03 ± 0.06cdefg	2.53 ± 0.03cde	4.57 ± 0.10 cd
	R3	49.00 ± 1.35de	46.42 ± 1.78fg	95.43 ± 3.13ef	0.98 ± 0.01	2.62 ± 0.03b	3.60 ± 0.04b	2.83 ± 0.45ghi	7.11 ± 0.26d	9.94 ± 0.71f	1.72 ± 0.07efgh	2.82 ± 0.08cd	4.53 ± 0.15d
mean rye		***53.14 ± 11.48***	***59.89 ± 12.40***	***113.03 ± 16.64***	***1.14 ± 0.16***	***3.08 ± 0.48***	***4.22 ± 0.57***	***2.96 ± 0.54***	***9.23 ± 1.88***	***12.19 ± 1.96***	***1.83 ± 0.17***	***2.77 ± 0.22***	***4.61 ± 0.10***
barley	B1	40.13 ± 2.45fg	36.82 ± 1.46ghi	76.95 ± 3.9gh	2.13 ± 0.14bcde	2.49 ± 0.13b	4.63 ± 0.27b	5.12 ± 0.1de	5.62 ± 0.25def	10.74 ± 0.36f	2.34 ± 0.04bcde	2.34 ± 0.03def	4.68 ± 0.07cd
	B2	48.02 ± 3.85de	41.33 ± 1.35fgh	89.35 ± 5.21fg	2.06 ± 0.04bcde	1.84 ± 1.11b	3.91 ± 1.15b	4.11 ± 0.53ef	5.88 ± 0.25de	9.99 ± 0.78f	2.23 ± 0.26bcdef	2.18 ± 0.35efg	4.41 ± 0.61de
	B3	54.23 ± 2.98cd	10.77 ± 0.69k	64.00 ± 3.67ij	2.64 ± 0.05bcde	2.34 ± 0.06b	4.98 ± 0.11b	5.68 ± 0.28d	2.19 ± 0.02i	7.88 ± 0.30g	3.09 ± 0.10b	1.67 ± 0.06gh	4.75 ± 0.16cd
mean barley		***47.46 ± 7.07***	***29.64 ± 16.50***	***77.10 ± 12.18***	***2.28 ± 0.32***	***2.22 ± 0.34***	***4.50 ± 0.55***	***4.97 ± 0.80***	***4.57 ± 2.06***	***9.54 ± 1.48***	***2.55 ± 0.46***	***2.06 ± 0.35***	***4.61 ± 0.18***
pearl millet	M1	58.70 ± 3.68c	94.35 ± 3.03b	153.05 ± 6.71b	3.21 ± 0.00bc	4.37 ± 0.03b	7.58 ± 0.04b	8.51 ± 0.45c	16.19 ± 0.44b	24.70 ± 0.89b	2.91 ± 0.29b	1.79 ± 0.04fgh	4.71 ± 0.33cd
	M2	18.44 ± 0.55ij	67.60 ± 2.66d	86.04 ± 3.21fg	1.75 ± 0.04bcde	3.69 ± 0.10b	5.43 ± 0.13b	3.54 ± 0.13fgh	11.07 ± 0.16c	14.61 ± 0.29e	1.59 ± 0.03efgh	2.87 ± 0.01cd	4.45 ± 0.04d
mean millet		***38.57 ± 28.47***	***80.97 ± 18.91***	***119.54 ± 47.38***	***2.48 ± 1.04***	***4.03 ± 0.48***	***6.51 ± 1.52***	***6.03 ± 3.51***	***13.63 ± 3.63***	***19.66 ± 7.14***	***2.25 ± 0.94***	***2.33 ± 0.76***	***4.58 ± 0.18***
wheat	W1	21.05 ± 0.93ijk	27.48 ± 1.36ij	48.52 ± 2.29jk	0.85 ± 0.08de	1.74 ± 0.07b	2.59 ± 0.16b	1.39 ± 0.22kl	3.09 ± 0.10de	4.48 ± 0.33hi	1.40 ± 0.09fgh	1.63 ± 0.09gh	3.02 ± 0.18fg
	W2	22.87 ± 0.77hi	31.68 ± 0.86hij	54.54 ± 1.63ijk	1.33 ± 0.01cde	2.36 ± 0.06b	3.69 ± 0.07b	1.93 ± 0.14ijk	4.48 ± 0.02efg	6.41 ± 0.15gh	1.54 ± 0.09efgh	1.41 ± 0.00h	2.96 ± 0.10fg
	W3	26.85 ± 1.03h	38.52 ± 1.51ghi	65.37 ± 2.53hi	1.14 ± 0.05cde	1.94 ± 0.05b	3.09 ± 0.10b	2.02 ± 0.50ijk	4.23 ± 0.06fgh	6.25 ± 0.56gh	1.30 ± 0.48gh	1.87 ± 0.02fgh	3.18 ± 0.51fg
	W4	18.64 ± 1.93j	28.44 ± 1.31ij	47.09 ± 3.23k	1.07 ± 0.04cde	1.50 ± 0.04b	2.57 ± 0.09b	1.44 ± 0.59jkl	3.24 ± 0.06ghi	4.68 ± 0.65hi	1.19 ± 0.03h	1.80 ± 0.02gh	2.99 ± 0.06g
	W5	15.02 ± 0.59j	25.79 ± 3.88ij	40.81 ± 4.47k	3.60 ± 0.02b	1.66 ± 0.03b	5.25 ± 0.05b	1.87 ± 0.50jkl	3.55 ± 0.02ghi	5.41 ± 0.52hi	1.05 ± 0.01h	1.65 ± 0.04gh	2.70 ± 0.06g
mean wheat		***20.88 ± 4.44***	***30.38 ± 5.03***	***51.27 ± 9.27***	***1.60 ± 1.13***	***1.84 ± 0.33***	***3.44 ± 1.11***	***1.73 ± 0.29***	***3.72 ± 0.61***	***5.45 ± 0.88***	***1.30 ± 0.19***	***1.67 ± 0.18***	***2.97 ± 0.17***
triticale	T1	36.23 ± 1.17g	30.52 ± 0.75hij	66.75 ± 1.92hi	1.09 ± 0.24cde	1.90 ± 0.02b	2.99 ± 0.26b	1.88 ± .026ijk	5.34 ± 0.37ghi	7.22 ± 0.64g	1.46 ± 0.04fgh	2.20 ± 0.08efg	3.66 ± 0.11ef
	T2	22.94 ± 1.69hi	21.26 ± 1.09jk	44.21 ± 2.78jk	0.71 ± 0.19e	1.29 ± 0.04b	2.00 ± 0.23b	0.76 ± 0.06l	2.72 ± 0.22hi	3.48 ± 0.28i	1.50 ± 0.08efgh	1.47 ± 0.09h	2.97 ± 0.18fg
	T3	36.19 ± 1.52g	21.69 ± 0.15jk	57.88 ± 1.67ij	1.13 ± 0.10cde	1.36 ± 0.04b	2.48 ± 0.13b	2.24 ± 0.29ijk	2.92 ± 0.09ghi	5.16 ± 0.38hi	1.76 ± 0.03efgh	1.58 ± 0.00h	3.33 ± 0.04fg

mean triticale		***31.79 ± 7.66***	***24.49 ± 5.22***	***56.28 ± 11.36***	***0.98 ± 0.23***	***1.51 ± 0.33***	***2.49 ± 0.50***	***1.63 ± 0.77***	***3.66 ± 1.46***	***5.29 ± 1.87***	***1.57 ± 0.16***	***1.75 ± 0.39***	***3.32 ± 0.34***
sorghum	S1	21.02 ± 1.02hij	82.17 ± 10.86c	103.18 ± 11.88de	2.42 ± 0.12bcde	5.24 ± 0.31b	7.66 ± 0.43b	3.89 ± 0.28f	16.01 ± 0.81b	19.90 ± 1.09c	1.85 ± 0.25defgh	9.68 ± 0.52b	11.53 ± 0.77b
	S2	49.84 ± 0.94de	61.79 ± 1.16de	111.63 ± 2.10d	2.99 ± 0.04bcd	3.47 ± 0.20b	6.45 ± 0.24b	7.77 ± 0.25c	10.29 ± 0.31c	18.05 ± 0.56cd	2.71 ± 0.09bcd	1.73 ± 0.05gh	4.45 ± 0.14d
	S3	165.98 ± 1.03a	191.65 ± 10.95a	357.62 ± 11.98a	37.93 ± 2.46a	153.54 ± 25.58a	191.47 ± 28.04a	29.96 ± 0.27a	31.33 ± 1.81a	61.29 ± 2.08a	12.78 ± 1.04a	17.11 ± 0.53a	29.89 ± 1.57a
	S4	53.80 ± 1.38cd	51.57 ± 1.26ef	105.37 ± 2.65de	3.73 ± 0.21b	2.54 ± 0.32b	6.27 ± 0.53b	10.17 ± 0.33b	6.99 ± 0.20d	17.16 ± 0.52d	2.74 ± 0.13bc	2.58 ± 0.06cde	5.32 ± 0.19c
mean sorghum		***72.66 ± 63.90***	***96.79 ± 64.50***	***169.45 ± 125.50***	***11.77 ± 17.45***	***41.20 ± 74.90***	***52.96 ± 92.34***	***12.95 ± 11.63***	***16.15 ± 10.78***	***29.10 ± 21.49***	***5.02 ± 5.19***	***7.77 ± 7.17***	***12.80 ± 11.82***
**mean flour**		**44.08 ± 18.05**	**53.69 ± 30.38**	**97.78 ± 45.07**	**3.37 ± 4.15**	**8.98 ± 15.81**	**12.35 ± 19.94**	**5.04 ± 4.25**	**8.49 ± 5.43**	**13.54 ± 9.29**	**2.42 ± 1.35**	**3.06 ± 2.34**	**5.48 ± 3.66**

gen = genotype. Data presented are means ± standard deviation (n=3). Different letters in each column indicate significant differences according to Tukey's test ($p < 0.05$). Bold values are the mean values (mean ± SD) of cereal species.

Supplementary Table S3. Total phenolic content and antioxidant capacity of wholegrain flours.

wholegrain flours		TPC (mg GAE/100 g dw)			DPPH (µmol TE/g dw)			FRAP (µmol TE/g dw)			ABTS (µmol TE/g dw)		
**cereal**	**gen**	**free**	**bound**	**total**	**free**	**bound**	**total**	**free**	**bound**	**total**	**free**	**bound**	**total**
oat	O1	31.32 ± 1.95jkl	83.80 ± 5.30kl	115.11 ± 7.25klm	1.23 ± 0.11b	3.08 ± 0.12b	4.31 ± 0.24c	4.86 ± 0.46cde	16.05 ± 0.70fg	20.91 ± 1.15efg	2.29 ± 0.40c	3.28 ± 0.04de	5.57 ± 0.43cd
	O2	19.48 ± 0.31m	47.13 ± 2.77o	66.61 ± 3.08p	0.76 ± 0.04b	1.91 ± 0.17b	2.67 ± 0.21c	2.13 ± 0.99cde	9.17 ± 0.59h	11.31 ± 0.68g	1.33 ± 0.01c	3.02 ± 0.18e	4.35 ± 0.18d
	O3	58.54 ± 0.88e	71.68 ± 1.09m	130.22 ± 1.97ij	1.96 ± 0.04b	2.67 ± 0.08b	4.62 ± 0.14c	7.81 ± 0.41cde	14.55 ± 0.98gh	22.36 ± 1.39defg	2.97 ± 0.25c	3.31 ± 0.02de	6.29 ± 0.27cd
mean oat		***36.45 ± 20.03***	***67.54 ± 18.68***	***103.98 ± 33.23***	***1.32 ± 0.60***	***2.55 ± 0.59***	***3.87 ± 1.05***	***4.93 ± 2.84***	***13.26 ± 3.62***	***18.19 ± 6.01***	***2.20 ± 0.82***	***3.20 ± 0.16***	***5.40 ± 0.98***
rye	R1	47.40 ± 2.46fgh	132.87 ± 3.80c	180.27 ± 6.26e	1.47 ± 0.11b	4.20 ± 0.23b	5.67 ± 0.33c	5.88 ± 0.67cde	26.28 ± 1.24c	32.16 ± 1.91bcd	3.04 ± 0.11c	16.10 ± 0.49c	19.14 ± 0.60cd
	R2	44.55 ± 1.62h	82.66 ± 2.25l	127.21 ± 3.87ijk	1.56 ± 0.11b	3.21 ± 0.03b	4.77 ± 0.15c	5.36 ± 0.40cde	18.33 ± 1.69defg	23.69 ± 2.08defg	3.12 ± 0.15c	12.22 ± 0.58c	15.35 ± 0.73cd
	R3	47.18 ± 2.04gh	113.03 ± 0.80de	160.21 ± 2.83ff	1.68 ± 0.02b	3.82 ± 0.11b	5.50 ± 0.13c	5.78 ± 0.33cde	22.91 ± 0.37cde	28.68 ± 0.70def	3.65 ± 0.02c	14.55 ± 0.61c	18.20 ± 0.63cd
mean rye		***46.38 ± 1.58***	***109.52 ± 25.29***	***155.89 ± 26.79***	***1.57 ± 0.10***	***3.74 ± 0.50***	***5.31 ± 0.48***	***5.67 ± 0.27***	***22.51 ± 3.99***	***28.18 ± 4.26***	***3.27 ± 0.33***	***14.29 ± 1.95***	***17.56 ± 1.97***
barley	B1	56.37 ± 2.69e	107.42 ± 2.99ef	163.79 ± 5.68f	3.90 ± 0.29b	3.52 ± 0.25b	7.42 ± 0.54c	11.03 ± 0.86cd	18.16 ± 0.56defg	29.19 ± 1.42cdef	3.73 ± 0.03c	3.19 ± 0.08de	6.92 ± 0.11cd
	B2	42.02 ± 0.14hi	95.33 ± 3.28hij	137.36 ± 3.42hi	2.95 ± 0.27b	3.32 ± 0.09b	6.27 ± 0.36c	7.67 ± 0.68cde	17.06 ± 1.09efg	24.73 ± 1.77def	3.47 ± 0.33c	12.68 ± 0.29c	16.15 ± 0.62cd
	B3	54.16 ± 1.71ef	97.01 ± 3.59ghi	151.17 ± 5.29fg	2.65 ± 0.14b	3.34 ± 0.02b	5.99 ± 0.17c	7.42 ± 0.50cde	18.44 ± 1.61defg	25.87 ± 2.11def	3.03 ± 0.11c	12.82 ± 0.61c	15.85 ± 0.72cd
mean barley		***50.85 ± 7.72***	***99.92 ± 6.55***	***150.77 ± 13.22***	***3.16 ± 0.65***	***3.39 ± 0.11***	***6.56 ± 0.76***	***8.71 ± 2.02***	***17.89 ± 0.73***	***26.60 ± 2.32***	***3.41 ± 0.35***	***9.56 ± 5.52***	***12.97 ± 5.25***
pearl millet	M1	67.86 ± 1.21d	164.03 ± 3.07b	231.89 ± 4.28c	3.02 ± 0.08b	4.59 ± 0.23b	7.61 ± 0.31c	9.22 ± 0.69cde	65.87 ± 4.82c	75.09 ± 5.51b	2.07 ± 0.11c	3.11 ± 0.03e	5.18 ± 0.14cd
	M2	40.82 ± 1.01hi	119.85 ± 1.91d	160.66 ± 2.92f	2.45 ± 0.06b	4.27 ± 0.24b	6.72 ± 0.30c	6.22 ± 0.57cde	25.29 ± 0.54c	31.51 ± 1.11cdef	2.32 ± 0.07c	3.12 ± 0.05e	5.44 ± 0.12cd
mean millet		***54.34 ± 19.12***	***141.94 ± 31.24***	***196.28 ± 50.36***	***2.73 ± 0.40***	***4.43 ± 0.23***	***7.16 ± 0.63***	***7.72 ± 2.12***	***45.58 ± 28.69***	***53.30 ± 30.81***	***2.19 ± 0.17***	***3.12 ± 0.01***	***5.31 ± 0.18***
wheat	W1	28.51 ± 0.80l	103.38 ± 7.20fgh	131.89 ± 8.01ij	1.24 ± 0.20b	4.47 ± 0.14b	5.71 ± 0.34c	3.17 ± 0.07cde	24.11 ± 0.40cd	27.28 ± 0.48def	1.90 ± 0.10c	13.24 ± 0.21c	15.14 ± 0.31cd
	W2	26.71 ± 1.80 l	95.80 ± 2.34hi	122.50 ± 4.13jkl	1.37 ± 0.08b	4.14 ± 0.09b	5.51 ± 0.17c	3.75 ± 0.10cde	22.02 ± 1.98	25.78 ± 2.08def	1.98 ± 0.22c	12.34 ± 0.09c	14.31 ± 0.31cd
	W3	30.73 ± 2.02jkl	72.67 ± 1.80m	103.40 ± 3.82mno	1.27 ± 0.12b	3.99 ± 0.08b	5.26 ± 0.20c	1.96 ± 0.91cde	18.07 ± 0.48defg	20.03 ± 1.40efg	1.41 ± 0.12c	11.61 ± 0.38c	13.03 ± 0.50cd
	W4	37.55 ± 0.93ij	56.10 ± 1.73n	93.65 ± 2.67o	1.27 ± 0.06b	3.21 ± 0.05b	4.48 ± 0.11c	2.89 ± 0.34cde	16.71 ± 1.27efg	19.60 ± 1.60efg	1.41 ± 0.35c	1.94 ± 0.10e	3.35 ± 0.45d
	W5	53.71 ± 1.59efg	92.26 ± 2.04ijk	145.97 ± 3.63gh	1.46 ± 0.04b	4.49 ± 0.04b	5.95 ± 0.08c	3.59 ± 0.21cde	22.98 ± 2.43cde	26.57 ± 2.64def	2.87 ± 0.15c	13.47 ± 0.63c	16.35 ± 0.78cd

mean wheat		***35.44 ± 11.01***	***84.04 ± 19.30***	***119.48 ± 21.26***	***1.32 ± 0.09***	***4.06 ± 0.52***	***5.38 ± 0.56***	***3.07 ± 0.71***	***20.78 ± 3.22***	***23.85 ± 3.73***	***1.92 ± 0.60***	***10.52 ± 4.85***	***12.44 ± 5.22***
triticale	T1	30.58 ± 0.93kl	71.42 ± 1.26m	101.99 ± 2.19no	1.48 ± 0.11b	3.19 ± 0.20b	4.67 ± 0.30c	3.93 ± 0.36cde	16.76 ± 1.44efg	20.69 ± 1.80efgd	1.81 ± 0.16c	2.10 ± 0.10e	3.91 ± 0.26d
	T2	26.71 ± 0.28l	84.42 ± 1.31kl	111.12 ± 1.59lmn	1.27 ± 0.09b	3.54 ± 0.10b	4.81 ± 0.19c	3.94 ± 0.38cde	17.75 ± 0.55def	21.69 ± 0.93defg	1.98 ± 0.05c	12.78 ± 0.33c	14.76 ± 0.38cd
	T3	36.73 ± 1.13ijk	58.36 ± 0.84n	95.09 ± 1.97o	1.32 ± 0.15b	2.89 ± 0.24b	4.20 ± 0.39c	4.01 ± 0.02cde	14.66 ± 0.64gh	18.67 ± 0.66fg	2.24 ± 0.07c	3.10 ± 0.06e	5.34 ± 0.13cd
mean triticale		***31.34 ± 5.06***	***71.40 ± 13.03***	***102.73 ± 8.04***	***1.35 ± 0.11***	***3.20 ± 0.33***	***4.56 ± 0.32***	***3.96 ± 0.04***	***16.39 ± 1.58***	***20.35 ± 1.54***	***2.01 ± 0.22***	***5.99 ± 5.90***	***8.00 ± 5.90***
sorghum	S1	220.97 ± 5.96b	349.84 ± 2.50a	570.80 ± 8.45b	98.85 ± 86.72b	128.73 ± 22.75b	227.58 ± 109.47a	201.11 ± 10.60a	113.31 ± 3.40a	[illegible]14.42 ± 14.00a	143.71 ± 13.86b	94.86 ± 3.04b	238.57 ± 16.89b
	S2	125.29 ± 1.87c	86.39 ± 2.88jkl	211.68 ± 4.75d	58.15 ± 24.71b	3.31 ± 0.05b	61.46 ± 24.76b	20.58 ± 0.79b	21.66 ± 1.83cdef	42.23 ± 2.62c	14.07 ± 0.00c	15.59 ± 0.30c	29.66 ± 0.30c
	S3	290.49 ± 3.88a	356.51 ± 3.20a	647.00 ± 7.08a	86.29 ± 31.24a	145.88 ± 11.10b	232.17 ± 42.34a	204.39 ± 6.51a	115.74 ± 6.31a	[illegible]20.12 ± 12.82a	176.40 ± 21.73a	111.15 ± 12.47a	287.54 ± 21.88a
	S4	56.91 ± 3.59e	105.48 ± 2.65efg	162.39 ± 6.24f	4.28 ± 0.06a	4.12 ± 0.05b	8.40 ± 0.11c	11.31 ± 0.50cd	22.79 ± 1.13cde	34.13 ± 1.64cd	7.90 ± 0.86c	17.38 ± 0.15c	25.28 ± 0.86cd
mean sorghum		***173.41 ± 103.05***	***224.56 ± 148.75***	***397.97 ± 246.37***	***61.89 ± 42.01***	***70.51 ± 77.45***	***132.40 ± 114.63***	***109.35 ± 107.92***	***68.37 ± 53.30***	***[illegible]77.73 ± 161.18***	***85.52 ± 87.13***	***59.75 ± 50.40***	***145.26 ± 137.49***
mean wholegrain flour		***61.17 ± 50.22***	***114.13 ± 54.91***	***175.30 ± 103.66***	***10.48 ± 22.68***	***13.13 ± 25.31***	***23.61 ± 47.99***	***20.49 ± 39.24***	***29.25 ± 20.27***	***49.74 ± 57.62***	***14.36 ± 31.38***	***15.21 ± 20.06***	***29.56 ± 51.21***

gen = genotype. Data presented are means ± standard deviation (n=3). Different letters in each column indicate significant differences according to Tukey's test ($p < 0.05$). Bold values are the mean values (mean ± SD) of cereal species.

Supplementary Table S4. Total phenolic content and antioxidant capacity of bran samples.

bran cereal crop	gen	TPC (mg GAE/100 g dw)			DPPH (µmol TE/g dw)			FRAP (µmol TE/g dw)			ABTS (µmol TE/g dw)		
		free	bound	total	free	bound	total	free	bound	total	free	bound	total
rye	R1	77.03 ± 1.50fgh	150.38 ± 5.11kl	227.41 ± 6.61fg	4.07 ± 0.24d	34.24 ± 6.04cd	38.30 ± 6.28e	13.63 ± 0.16fgh	494.63 ± 14.31a	508.25 ± 14.47b	26.74 ± 0.20cd	26.47 ± 2.65f	53.21 ± 2.85def
	R2	96.83 ± 2.48efgh	38.54 ± 0.35m	135.37 ± 2.83h	4.02 ± 0.22d	40.96 ± 6.60cd	44.98 ± 6.82e	12.72 ± 0.73fgh	8.35 ± 0.63ij	21.07 ± 1.35hi	12.4 ± 2.53cd	57.03 ± 1.00ef	69.51 ± 3.53def
	R3	98.61 ± 2.82defgh	159.53 ± 5.46ijk	258.14 ± 8.28efg	4.30 ± 0.31d	26.08 ± 1.10d	30.39 ± 1.41e	13.91 ± 0.49fgh	2.90 ± 2.18j	16.81 ± 2.66hi	16.67 ± 2.49cd	38.75 ± 3.04ef	55.41 ± 5.53def
mean rye		***90.82 ± 11.98***	***116.15 ± 67.37***	***206.97 ± 63.89***	***4.13 ± 0.15***	***33.76 ± 7.45***	***37.89 ± 7.31***	***13.42 ± 0.62***	***168.63 ± 282.34***	***182.04 ± 282.51***	***18.63 ± 7.33***	***40.75 ± 15.38***	***59.38 ± 8.84***
barley	B1	116.30 ± 3.54cdefgh	144.79 ± 1.66kl	261.09 ± 5.19efg	147.87 ± 16.64c	32.26 ± 1.97cd	180.13 ± 18.60d	27.74 ± 0.94cd	26.88 ± 0.39ghi	54.62 ± 1.34f	23.92 ± 5.45cd	48.33 ± 9.05ef	72.25 ± 14.50def
	B2	126.90 ± 1.68cdefg	200.86 ± 6.54gh	327.76 ± 8.22e	55.81 ± 11.04d	34.70 ± 2.75cd	90.51 ± 13.79e	31.88 ± 0.34c	61.64 ± 11.36de	93.53 ± 11.70d	24.35 ± 5.64cd	44.71 ± 6.93ef	69.06 ± 12.56def
	B3	83.92 ± 2.85fgh	157.02 ± 1.59jkl	240.94 ± 4.43efg	6.75 ± 0.11d	37.87 ± 1.04cd	44.62 ± 1.15e	17.56 ± 1.52efg	35.24 ± 13.68fgh	52.80 ± 15.20f	13.20 ± 0.79cd	45.01 ± 8.66ef	58.21 ± 9.45def
mean barley		***109.04 ± 22.39***	***167.56 ± 29.48***	***276.60 ± 45.44***	***70.14 ± 71.64***	***34.94 ± 2.81***	***105.09 ± 68.92***	***25.73 ± 7.37***	***41.26 ± 18.15***	***66.98 ± 23.01***	***20.49 ± 6.32***	***46.02 ± 2.01***	***66.51 ± 7.36***
pearl millet	M1	179.03 ± 3.25c	404.29 ± 12.99c	583.32 ± 16.24c	179.21 ± 46.02c	102.30 ± 4.55b	281.51 ± 50.57c	21.44 ± 0.56def	67.73 ± 0.33d	89.17 ± 0.90de	10.73 ± 0.26cd	122.62 ± 0.46c	133.35 ± 0.73c
	M2	159.11 ± 5.34cde	296.17 ± 34.63d	455.29 ± 39.97d	136.96 ± 26.65c	79.83 ± 7.48bc	216.78 ± 34.13d	25.08 ± 2.36cde	48.56 ± 0.37def	73.65 ± 15.20e	10.10 ± 1.25cd	93.71 ± 8.15cd	103.81 ± 9.39cd
mean millet		***169.07 ± 14.08***	***350.23 ± 76.45***	***519.30 ± 90.53***	***158.08 ± 29.88***	***91.06 ± 15.89***	***249.15 ± 45.77***	***23.26 ± 2.57***	***58.14 ± 13.55***	***81.41 ± 10.98***	***10.42 ± 0.44***	***108.16 ± 20.45***	***118.58 ± 20.89***
wheat	W1	67.02 ± 2.61fgh	206.78 ± 5.13gh	273.80 ± 7.74ef	4.95 ± 0.18d	45.50 ± 4.29cd	50.45 ± 4.48e	10.48 ± 0.24ghi	12.53 ± 0.75ij	23.01 ± 0.99hi	3.91 ± 2.03d	46.80 ± 0.45ef	50.71 ± 2.49ef
	W2	61.46 ± 1.47fgh	265.48 ± 7.28de	326.94 ± 8.74e	5.84 ± 2.60d	61.35 ± 9.04bcd	67.18 ± 11.64e	8.94 ± 0.09ghi	20.98 ± 0.88hij	29.92 ± 0.99hi	2.91 ± 0.06d	42.65 ± 2.42ef	45.56 ± 2.48ef
	W3	54.84 ± 1.90gh	209.32 ± 25.62g	264.16 ± 27.52efg	2.91 ± 0.16d	69.01 ± 12.97bcd	71.92 ± 13.13e	3.29 ± 0.15ij	18.33 ± 0.28hij	21.62 ± 0.43hi	2.71 ± 1.51d	47.86 ± 7.70ef	50.57 ± 9.21
	W4	96.72 ± 2.96defgh	217.52 ± 8.21fg	314.24 ± 11.17ef	4.45 ± 0.21d	46.30 ± 6.02cd	50.75 ± 6.22e	8.08 ± 0.13hij	14.39 ± 0.72ij	22.47 ± 0.85hi	5.42 ± 0.19d	51.46 ± 1.34ef	56.89 ± 1.53def
	W5	54.60 ± 3.07gh	123.00 ± 4.50l	177.60 ± 7.57h	3.85 ± 0.28d	70.11 ± 5.43bcd	73.96 ± 5.71e	5.85 ± 1.86hij	6.48 ± 0.24ij	12.33 ± 2.10i	9.29 ± 1.66cd	37.55 ± 1.84ef	46.84 ± 3.50ef
mean wheat		***66.93 ± 17.43***	***204.42 ± 51.38***	***271.35 ± 58.69***	***4.40 ± 1.11***	***58.45 ± 11.95***	***62.85 ± 11.45***	***7.33 ± 2.81***	***14.54 ± 5.59***	***21.87 ± 6.28***	***4.85 ± 2.71***	***45.26 ± 5.34***	***50.11 ± 4.41***
triticale	T1	77.54 ± 1.64fgh	223.31 ± 5.31fg	300.85 ± 6.95ef	3.06 ± 0.13d	51.81 ± 5.44cd	54.88 ± 5.57e	7.50 ± 0.22hij	9.25 ± 1.52ij	16.76 ± 1.74hi	12.36 ± 1.52cd	54.53 ± 5.95ef	66.89 ± 7.47def
	T2	61.42 ± 2.44fgh	194.23 ± 3.30ghi	255.65 ± 5.74efg	2.24 ± 0.11d	53.58 ± 4.64cd	55.82 ± 4.75e	7.06 ± 0.32hij	7.16 ± 1.83ij	14.22 ± 2.15hi	11.92 ± 1.59cd	57.99 ± 5.48ef	69.90 ± 7.07def
	T3	48.63 ± 1.70h	188.63 ± 1.35ghij	237.26 ± 3.05fg	2.88 ± 0.11d	49.96 ± 6.71cd	52.84 ± 6.82e	6.68 ± 0.17hij	7.52 ± 0.92ij	14.19 ± 1.10hi	11.68 ± 11.74cd	22.90 ± 5.97f	34.58 ± 7.71f

mean triticale		***62.53 ± 14.49***	***202.06 ± 18.62***	***264.59 ± 32.72***	***2.73 ± 0.43***	***51.79 ± 1.81***	***54.51 ± 1.52***	***7.08 ± 0.41***	***7.98 ± 1.12***	***15.05 ± 1.47***	***11.98 ± 0.35***	***45.14 ± 19.33***	***57.12 ± 19.58***
sorghum	S1	1184.25 ± 50.88b	791.69 ± 17.00b	1975.94 ± 67.89b	421.46 ± 55.00b	280.39 ± 54.76a	701.85 ± 109.77b	349.45 ± 4.83b	262.30 ± 4.59b	611.76 ± 9.42b	319.79 ± 32.37b	285.66 ± 39.94b	605.45 ± 72.31b
	S2	170.17 ± 8.88cd	250.00 ± 6.57ef	420.17 ± 15.46d	138.71 ± 24.03c	44.30 ± 2.54cd	183.02 ± 26.56c	1.26 ± 0.61ij	51.50 ± 4.38def	52.76 ± 4.99f	37.49 ± 3.06c	59.56 ± 3.43de	97.05 ± 6.49cde
	S3	1800.88 ± 91.46a	1024.63 ± 8.07a	2825.51 ± 99.53a	519.98 ± 15.70a	286.93 ± 12.68a	806.91 ± 28.41a	414.88 ± 0.61a	224.17 ± 16.93c	639.05 ± 28.81a	373.50 ± 20.84a	348.33 ± 4.27a	721.83 ± 25.11a
	S4	130.38 ± 5.65cdef	171.19 ± 2.73hijk	301.57 ± 8.38ef	135.99 ± 16.97c	44.07 ± 4.77cd	180.06 ± 21.73c	0.28 ± 0.22j	42.77 ± 0.74efg	43.05 ± 0.96fg	12.38 ± 3.11cd	38.33 ± 4.27ef	50.83 ± 3.33ef
mean sorghum		***821.42 ± 814.99***	***559.38 ± 415.06***	***1380.80 ± 1228.67***	***304.04 ± 196.63***	***163.92 ± 138.28***	***467.96 ± 333.50***	***191.47 ± 221.81***	***145.18 ± 114.34***	***336.65 ± 333.63***	***185.79 ± 187.31***	***183.00 ± 157.06***	***368.79 ± 344.28***
mean bran		***219.97 ± 297.16***	***266.63 ± 163.21***	***486.60 ± 451.35***	***90.59 ± 120.96***	***72.32 ± 49.48***	***162.91 ± 168.06***	***44.71 ± 72.32***	***72.62 ± 68.16***	***117.34 ± 123.04***	***42.03 ± 70.66***	***78.05 ± 57.44***	***120.08 ± 124.32***

gen = genotype. Data presented are means ± standard deviation (n=3). Different letters in each column indicate significant differences according to Tukey's test ($p < 0.05$). Bold values are the mean values (mean ± SD) of cereal species.

Supplementary Table S5. Total phenolic content and antioxidant capacity of husk samples.

husk		TPC (mg GAE/100 g dw)			DPPH (umol TE/g dw)			FRAP (umol TE/g dw)			ABTS (umol TE/g dw)		
**cereal**	**gen**	**free**	**bound**	**total**	**free**	**bound**	**total**	**free**	**bound**	**total**	**free**	**bound**	**total**
oat	O1	23.97 ± 0.88d	274.49 ± 12.10ab	298.46 ± 12.98ab	1.32 ± 0.04c	79.86 ± 1.07a	81.18 ± 1.11a	2.82 ± 1.77d	65.05 ± 30.52a	67.86 ± 32.28a	7.99 ± 2.31a	89.87 ± 7.75a	97.86 ± 10.06a
	O2	31.26 ± 1.44c	233.67 ± 33.54bc	264.93 ± 34.98bc	1.50 ± 0.15c	34.70 ± 1.02cd	36.20 ± 1.18d	5.43 ± 0.41cd	47.55 ± 8.99a	52.98 ± 9.41a	6.31 ± 0.42a	75.80 ± 6.89ab	82.11 ± 11.37ab
	O3	29.07 ± 0.38c	199.09 ± 11.97cd	228.15 ± 12.34c	1.25 ± 0.35c	45.10 ± 6.82b	46.36 ± 7.17c	3.24 ± 0.52d	54.53 ± 11.69a	57.77 ± 12.20a	7.27 ± 1.20a	76.31 ± 10.17ab	83.58 ± 11.37ab
media oat		***28.10 ± 3.74***	***235.75 ± 37.74***	***263.85 ± 35.17***	***1.36 ± 0.13***	***53.22 ± 23.65***	***54.58 ± 23.59***	***3.83 ± 1.40***	***55.71 ± 8.81***	***59.54 ± 7.60***	***7.19 ± 0.84***	***80.66 ± 7.98***	***87.85 ± 8.70***
barley	B1	70.05 ± 1.34a	162.39 ± 5.16d	232.44 ± 6.50c	6.53 ± 0.21a	33.38 ± 3.70d	39.91 ± 3.91cd	14.79 ± 3.66ab	27.27 ± 2.40a	42.06 ± 6.06a	12.58 ± 0.65a	38.12 ± 1.76d	50.70 ± 2.41c
	B2	72.70 ± 3.55a	256.84 ± 5.30ab	329.54 ± 8.85a	6.42 ± 0.33a	51.52 ± 1.03b	57.94 ± 1.36b	16.12 ± 1.13a	37.94 ± 10.34a	54.06 ± 11.47a	11.31 ± 0.70a	52.44 ± 11.76cd	63.75 ± 12.46bc
	B3	58.65 ± 1.10b	278.52 ± 10.16a	337.17 ± 11.26a	2.31 ± 0.18b	43.61 ± 1.36bc	45.92 ± 1.54c	10.05 ± 0.55bc	45.85 ± 4.65a	55.91 ± 5.20a	9.53 ± 028a	64.42 ± 2.01bc	73.95 ± 2.30abc
media barley		***67.13 ± 7.47***	***232.58 ± 61.75***	***299.72 ± 58.39***	***5.08 ± 2.41***	***42.84 ± 9.09***	***47.92 ± 9.18***	***13.66 ± 3.19***	***37.02 ± 9.33***	***50.67 ± 7.52***	***11.14 ± 1.53***	***51.66 ± 13.16***	***62.80 ± 11.65***
media husk		**47.62 ± 22.02**	**234.17 ± 45.81**	**281.78 ± 47.37**	**3.22 ± 2.55**	**48.03 ± 17.00**	**51.25 ± 16.42**	**8.74 ± 5.82**	**46.36 ± 13.06**	**55.11 ± 8.32**	**9.16 ± 2.43**	**66.16 ± 18.63**	**75.33 ± 16.52**

gen = genotype. Data presented are means ± standard deviation (n=3). Different letters in each column indicate significant differences according to Tukey’s test (p< 0.05). Bold values are the mean values (mean ± SD) of cereal species.

Supplementary Table S6. Discrimination of each annotated metabolite per sample.

No.	RT (min)	molecular formula	exp. $[M-H]^-$ *m/z*	proposed compound	cereal crop																				
					barley				pearl millet			oat		rye			sorghum			triticale			wheat		
					H	B	WG	F	B	WG	F	H	WG	B	WG	F	B	WG	F	B	WG	F	B	WG	F
Flavonoids																									
1	4.08	C15H12O7	303.0511	unknown flavonoid	x	x	x	-	x	-	-	x	x	x	x	x	x	x	x	x	x	-	x	x	x
2	5.05	C30H26O12	577.1390	procyanidin B-type	x	x	x	x	x	x	-	x	x	x	x	x	x	x	x	x	x	-	x	x	-
3	5.26	C15H10O7	301.0359	robinetin	x	x	x	x	x	x	x	x	x	x	x	x	x	x	x	x	x	-	x	x	x
4	5.29	C15H14O6	289.0722	luteoforol	x	x	x	x	x	x	-	x	x	x	x	x	x	x	x	x	x	-	x	x	x
5	**5.92**	C15H14O6	**289.0722**	**(-)-epicatechin**	x	x	x	x	x	x	x	x	x	x	x	x	x	x	x	x	x	-	x	x	x
6	6.00	C22H18O11	457.0810	epigallocatechin 3-gallate	x	x	x	-	x	x	-	x	x	x	x	x	x	x	x	x	x	-	x	x	x
7	6.11	C27H30O16	609.1519	saponarin	x	x	x	x	x	x	x	x	x	x	x	x	x	x	-	x	x	-	x	x	x
8	6.15	C46H44O18	883.2406	procyanidin derivative	x	x	x	-	x	x	-	x	x	-	x	x	x	x	x	x	-	-	x	x	x
9	6.38	C14H10O6	273.0402	anomalin A	x	x	x	x	x	x	x	x	x	x	x	x	x	x	x	x	x	x	x	x	x
10	6.60	C18H16O6	327.0884	betagarin	x	x	x	x	x	x	x	x	x	x	x	x	x	x	x	x	x	x	x	x	x
11	6.65	C15H10O5	269.0455	prunetol	x	x	x	x	x	x	x	x	x	x	x	x	x	x	x	x	x	x	x	x	x
12	6.78	C27H30O15	593.1563	isovitexin 2''-*O*-glucoside	x	x	x	x	x	x	x	x	x	x	x	x	x	x	x	x	x	x	x	x	x
13	6.90	C15H14O6	289.0719	catechin	x	x	x	x	x	x	x	x	x	x	x	x	x	x	x	x	x	x	x	x	x
14	6.96	C33H42O19	741.2275	narirutin 4-glucoside	x	x	x	x	x	x	x	x	x	x	x	x	x	x	x	x	x	x	x	x	x
15	7.13	C22H22O13	493.1007	isosakuranin	x	x	x	x	x	x	x	x	x	x	x	x	x	x	x	x	x	x	x	x	x
16	7.18	C22H18O10	441.0854	epicatechin 3-gallate	x	x	x	x	x	x	x	x	x	x	x	x	x	x	x	x	x	-	x	x	-
17	7.26	C22H26O11	465.1420	symplocoside	x	x	x	x	x	x	x	x	x	x	x	x	x	x	x	x	x	x	x	x	x
18	7.34	C16H12O5	283.0613	isoprunetin	x	x	x	x	x	x	x	x	x	x	x	x	x	x	x	x	x	x	x	x	x
19	7.42	C15H12O7	303.0518	unknown flavonoid	x	x	x	x	x	x	x	x	x	x	x	x	x	x	x	x	x	x	x	x	x
20	7.46	C26H30O14	565.1608	5,7,4'-trihydroxyflavanone 7-*O*-arabinosylglucoside	x	x	x	x	x	x	x	x	x	x	x	x	x	x	x	x	x	x	x	x	x
21	7.57	C15H12O7	303.0515	dihydroquercetin	x	x	x	x	x	x	x	x	x	x	x	x	x	x	x	x	x	x	x	x	x
22	7.89	C27H30O14	577.1592	flavonoid dissacharide	x	x	x	x	x	x	x	x	x	x	x	x	x	x	x	x	x	x	x	x	x
23	7.97	C36H34O16	721.1841	5-*O*-beta-D-glucosylluteoliflavan-(4->8)-eriodictyol	x	x	x	x	x	x	x	x	x	x	x	x	x	x	x	x	x	x	x	x	x
24	8.40	C16H14O6	301.0722	sterubin	x	x	x	x	x	x	x	x	x	x	x	x	x	x	x	x	x	x	x	x	x
25	**8.50**	**C15H10O8**	**317.0306**	**myricetin**	x	x	x	x	x	x	x	x	x	x	x	x	x	x	x	x	x	x	x	x	x
26	9.05	C21H20O9	415.1057	daidzin*	x	x	x	x	x	x	x	x	x	x	x	x	x	x	x	x	x	x	x	x	x
27	9.30	C21H20O9	415.1055	daidzin*	x	x	x	x	x	x	x	x	x	x	x	x	x	x	x	x	x	x	x	x	x
28	9.61	C15H12O6	287.0563	dihydrokaempferol	x	x	x	x	x	x	x	x	x	x	x	x	x	x	x	x	x	x	x	x	x
29	9.74	C15H10O6	285.0407	luteolin	x	x	x	x	x	x	x	x	x	x	x	x	x	x	x	x	x	x	x	x	x
30	9.92	C16H14O6	301.0722	flavonoid	x	x	x	x	x	x	x	x	x	x	x	x	x	x	x	x	x	x	x	x	x
31	10.08	C22H22O9	429.1213	ononin	x	x	x	x	x	x	x	x	x	x	x	x	x	x	x	x	x	x	x	x	x
32	10.54	C19H16O8	371.0787	quercetin 3-isobutyrate	x	x	x	x	x	x	x	x	x	x	x	x	x	x	x	x	x	x	x	x	x
33	10.84	C15H12O5	271.0615	naringenin	x	x	x	x	x	x	x	x	x	x	x	x	x	x	x	x	x	x	x	x	x
34	**10.88**	**C15H10O5**	**269.0454**	**apigenin**	x	x	x	x	x	x	x	x	x	x	x	x	x	x	x	x	x	x	x	x	x
35	10.98	C16H14O6	301.0717	flavonoid	x	x	x	x	x	x	x	x	x	x	x	x	x	x	x	x	x	x	x	x	x
36	11.06	C16H12O6	299.0563	flavonoid	x	x	x	x	x	x	x	x	x	x	x	x	x	x	x	x	x	x	x	x	x
37	**11.08**	**C15H10O6**	**285.0403**	**kaempferol**	x	x	x	x	x	x	x	x	x	x	x	x	x	x	x	x	x	x	x	x	x
38	11.10	C21H20O7	383.1150	gancaonin	x	x	x	x	x	x	x	x	x	x	x	x	x	x	x	x	x	x	x	x	x

No.	RT (min)	molecular formula	exp. $[M-H]^-$ *m/z*	proposed compound	cereal crop																				
					barley				pearl millet			oat		rye			sorghum			triticale			wheat		
					H	B	WG	F	B	WG	F	H	WG	B	WG	F	B	WG	F	B	WG	F	B	WG	F
phenolic acids and derivativ																									
39	3.89	C8H8O4	167.0338	dihydroxyphenylacetic acid	x	x	x	x	x	x	x	x	x	x	x	x	x	x	x	x	x	x	x	x	x
40	4.32	C7H6O4	153.0181	dihydroxybenzoic acid*	x	x	x	x	x	x	x	x	x	x	x	x	x	x	x	x	x	x	x	x	x
41	4.84	C9H10O4	181.0495	dihydrocaffeic acid	x	x	x	x	x	x	x	x	x	x	x	x	x	x	x	x	x	x	x	x	x
42	5.07	C15H18O9	341.0886	caffeoyl glucose	x	x	x	x	x	x	x	x	x	x	x	x	x	x	x	x	x	x	x	x	x
43	**5.19**	**C16H18O9**	**353.0887**	**chlorogenic acid**	x	x	x	x	x	x	x	x	x	x	x	x	x	x	x	x	x	x	x	x	x
44	**5.45**	**C7H6O3**	**137.0230**	**4-hydrozybenzoic acid**	x	x	x	x	x	x	x	x	x	x	x	x	x	x	x	x	x	x	x	x	x
45	**5.53**	C7H6O4	**153.0182**	**2,5 dihydroxybenzoic acid**	x	x	x	x	x	x	x	x	x	x	x	x	x	x	x	x	x	x	x	x	x
46	**5.88**	**C9H8O4**	**179.0341**	**caffeic acid**	x	x	x	x	x	x	x	x	x	x	x	x	x	x	x	x	x	x	x	x	x
47	5.98	C16H18O8	337.0939	coumaroylquinic acid	x	x	x	x	x	x	x	x	x	x	x	x	x	x	x	x	x	x	x	x	x
48	**6.00**	**C9H10O5**	**197.0446**	**syringic acid**	x	x	x	x	x	x	x	x	x	x	x	x	x	x	x	x	x	x	x	x	x
49	6.64	C17H20O9	367.1049	feruloylquinic acid	x	x	x	x	x	x	x	x	x	x	x	x	x	x	x	x	x	x	x	x	-
50	7.04	C9H8O3	163.0391	coumaric acid*	x	x	x	x	x	x	x	x	x	x	x	x	x	x	x	x	x	x	x	x	x
51	7.28	C20H22O8	389.1257	hydroxycinnamic acid derivative	x	x	x	x	x	x	x	x	x	x	x	x	x	x	-	x	x	x	x	x	x
52	**7.29**	C9H8O3	**163.0389**	***p*-coumaric acid**	x	x	x	x	x	x	x	x	x	x	x	x	x	x	x	x	x	x	x	x	x
53	**7.36**	**C11H12O5**	**223.0609**	**sinapic acid**	x	x	x	x	x	x	x	x	x	x	x	x	x	x	x	x	x	x	x	x	x
54	7.39	C20H18O8	385.0950	diferulic acid*	x	x	x	x	x	x	x	x	x	x	x	x	x	x	x	x	x	x	x	x	x
55	**7.40**	$\mathbf{C_{10}H_{10}O_4}$	**193.0501**	***trans*-ferulic acid**	x	x	x	x	x	x	x	x	x	x	x	x	x	x	x	x	x	x	x	x	x
56	7.56	C11H10O5	221.0450	*p*-coumaroyl glycolic acid	x	x	x	x	x	x	x	x	x	x	x	x	x	x	x	x	x	x	x	x	x
57	7.65	$C_{10}H_{10}O_4$	193.0500	ferulic acid*	x	x	x	x	x	x	x	x	x	x	x	x	x	x	x	x	x	x	x	x	x
58	7.81	C20H18O8	385.0950	diferulic acid*	x	x	x	x	x	x	x	x	x	x	x	x	x	x	x	x	x	x	x	x	x
59	8.41	C20H18O8	385.0946	diferulic acid*	x	x	x	x	x	x	x	x	x	x	x	x	x	x	x	x	x	x	x	x	x
60	8.71	C20H18O8	385.0947	diferulic acid*	x	x	x	x	x	x	x	x	x	x	x	x	x	x	x	x	x	x	x	x	x
61	8.92	C20H18O8	385.0949	diferulic acid*	x	x	x	x	x	x	x	x	x	x	x	x	x	x	x	x	x	x	x	x	x
62	5.34	C12H14O6	253.0716	2-*O*-caffeoylglycerol	x	x	x	x	x	x	x	x	x	x	x	x	x	x	x	x	x	x	x	x	x
63	7.44	C21H20O9	415.1055	1,3-*O*-dicaffeoylglycerol	x	x	x	x	x	x	x	x	x	x	x	x	x	x	x	x	x	x	x	x	x
64	9.45	C17H15NO6	328.0833	avenanthramide 2f	x	x	x	x	x	x	x	x	x	x	x	x	x	x	x	x	x	x	x	x	x
65	6.00	C25H31N3O6	468.2173	N(1),N(8)-bis(caffeoyl)spermidine	x	x	x	x	x	x	x	x	x	x	x	x	x	x	x	x	x	x	x	x	x
other polyphenols																									
66	5.73	C8H8O3	151.0387	vanillin*	x	x	x	x	x	x	x	x	x	x	x	x	x	x	x	x	x	x	x	x	x
67	6.57	C11H14O5	225.0762	isovanilmandelic acid ethyl ester	x	x	x	x	x	x	x	x	x	x	x	x	x	x	x	x	x	x	x	x	x
68	**7.00**	**C8H8O3**	**151.0387**	**vanillin**	x	x	x	x	x	x	x	x	x	x	x	x	x	x	x	x	x	x	x	x	x
69	8.90	C9H6O4	177.0180	aesculetin	x	x	x	x	x	x	x	x	x	x	x	x	x	x	x	x	x	x	x	x	x
lipid and lipid-like molecules																									
70	2.63	C6H10O5	161.0443	meglutol	x	x	x	x	x	x	x	x	x	x	x	x	x	x	x	x	x	x	x	x	x
71	8.47	C12H20O5	243.1235	fatty acid	x	x	x	x	x	x	x	x	x	x	x	x	x	x	x	x	x	x	x	x	x
72	8.58	C18H36O6	347.2451	sativic acid	x	x	x	x	x	x	x	x	x	x	x	x	x	x	x	x	x	x	x	x	x
72	8.90	C12H20O5	243.1235	oxododecanedioic acid	x	x	x	x	x	x	x	x	x	x	x	x	x	x	x	x	x	x	x	x	x
74	11.11	C20H38O7	389.2560	3-[(3,5-dihydroxydecanoyl)oxy]-5-hydroxydecanoic acid	x	x	x	x	x	x	x	x	x	x	x	x	x	x	x	x	x	x	x	x	x
75	12.38	C18H34O5	329.2343	fatty acid	x	x	x	x	x	x	x	x	x	x	x	x	x	x	x	x	x	x	x	x	x
76	12.54	C20H37O6; C19H36O4	373.2608	nonadecandioic acid	x	x	x	x	x	x	x	x	x	x	x	x	x	x	x	x	x	x	x	x	x

No.	RT (min)	molecular formula	exp. $[M-H]^-$ *m/z*	proposed compound	cereal crop																				
					barley				pearl millet			oat		rye			sorghum			triticale			wheat		
					H	B	WG	F	B	WG	F	H	WG	B	WG	F	B	WG	F	B	WG	F	B	WG	F
		[M+HCOO]- C19H36O4																							
organic acids																									
77	1.04	C6H12O7	195.0506	gulonic acid	x	x	x	x	x	x	x	x	x	x	x	x	x	x	x	x	x	x	x	x	x
78	1.33	C4H6O5	133.0130	malic acid	x	x	x	x	x	x	x	x	x	x	x	x	x	x	x	x	x	x	x	x	x
79	1.84	C6H8O7	191.0191	citric acid	x	x	x	x	x	x	x	x	x	x	x	x	x	x	x	x	x	x	x	x	x
80	5.05	C7H12O5	175.0604	isopropylmalic acid	x	x	x	x	x	x	x	x	x	x	x	x	x	x	x	x	x	x	x	x	x
other metabolites																									
81	3.05	C26H40O20	671.2106	oligosaccharide	x	x	x	x	x	x	x	x	x	x	x	x	x	x	x	x	x	x	x	x	x
82	1.97	C8H6O5	181.0131	4-(furan-2-yl)-2,4-dioxobutanoic acid	x	x	x	x	x	x	x	x	x	x	x	x	x	x	x	x	x	x	x	x	x
83	4.71	C11H12N2O2	203.0821	tryptophan	x	x	x	x	x	x	x	x	x	x	x	x	x	x	x	x	x	x	x	x	x
84	8.50	C35H44N8O4	639.3420	amino acid derivative	x	x	x	x	x	x	x	x	x	x	x	x	x	x	x	x	x	x	x	x	x
85	8.63	C23H27NO2	348.1939	robustinin	x	x	x	x	x	x	x	x	x	x	x	x	x	x	x	x	x	x	x	x	x
86	2.26	C9H12N2O6	243.0621	uridine	x	x	x	x	x	x	x	x	x	x	x	x	x	x	x	x	x	x	x	x	x
87	4.89	C12H12O7	267.0511	2-[(4-hydroxy-3,5-dimethoxyphenyl)methylidene]propanedioic acid	x	x	x	x	x	x	x	x	x	x	x	x	x	x	x	x	x	x	x	x	x

RT = retention time; exp., experimental; H = husk; WG = wholegrain flour; B = bran; F = flour.

Supplementary Table S7. List of the unknown metabolites highlighted by PLS-DA models.

No.	RT (min)	molecular formula	exp. [M-H]⁻ *m/z*	theoretical [M-H]- m/z	error (ppm)	level	MS/MS fragments (% relative intensity)	SIRIUS	VIP scores
1	3.31	unknown	465.9112	-	-	4	NF	x	2.220
2	6.03	unknown	284.1255	-	-	4	259.0617 (100), 209.0465 (30), 217.0491 (14)	x	3.216
3	6.06	C15H12O8	319.0463	319.0459	1.23	4	178.9996 (46), 227.0368 (46)	x	3.323[a]; 2.568[c]
4	6.46	unknown	656.1522	-	-	4	293.0465 (100), 593.1584 (70)	x	2.881
5	6.62	unknown	483.0959	-	-	4	125.0232 (100), 177.0193 (61), 123.0427 (54), 225.0757 (27), 299.0590 (24), 248.0322 (16)	x	2.382
4	7.33	C46H44O16	851.2503	851.2557	-6.25	4	NF	x	3.455
5	7.36	C17H12O6	311.0566	311.0561	1.62	4	NF	x	2.382
6	7.38	C20H22O7	373.1297	373.1293	1.10	4	243.0649 (56), 148.0375 (48), 256.0946 (45), 115.0029 (36)	x	3.589[c]; 2.285[d]
7	7.65	unknown	747.3472	-	-	4	747.3472 (100), 403.1030 (63), 385.0909 (59), 193.0466 (36), 134.0329 (24)	x	2.332
8	7.84	C22H30O15	533.1492	533.1512	-3.67	4	533.1492 (100), 489.1523 (40)	x	2.513
9	7.97	C11H8O4	203.0342	203.0350	-3.85	4	NF	x	3.029
10	9.52	C24H44O15	571.2668	571.2607	10.58	4	NF	x	2.336
11	9.74	C13H10O6	261.0396	261.0405	-3.30	4	158.0274 (100), 170.9097 (99), 217.0447 (93)	x	2.190
12	9.90	C14H8O6	271.0245	271.0248	-1.05	4	NF	x	2.931
13	10.30	unknown	343.0833	-	-	4	NF	x	2.020
14	10.62	C49H38O13	833.2221	833.2240	-2.21	4	NF	x	2.410
15	11.14	unknown	418.1232	-	-	4	329.2332 (100), 383.1138 (13)	x	2.669
16	11.14	unknown	490.0773	-	-	4	329.2348 (100)	x	2.821
17	11.66	unknown	376.1120	-	-	4	331.1915 (70), 287.2265 (15)	x	2.610
18	11.67	C14H8O6	271.0248	271.0248	-0.16	4	NF	x	2.198
19	12.88	C18H34O4	313.2393	313.2384	2.70	4	NF	x	2.564

NF, not fragmented; exp., experimental. Level of annotation: 1, compared to the authentic standards; 2, putatively annotation based on MS/MS spectral similarity with public libraries; 3, putatively class annotation based on the MS/MS spectral similarity to other annotated compounds and/or classified by the CANOPUS tool on SIRIUS software; 4, unknown metabolites. Letters in the VIP scores column mean: a,VIP in free extracts from cereal crops analysis; b, VIP in bound extracts from cereal crops analysis; c,VIP in free extracts from milling fractions analysis; d, VIP in bound extracts from milling fractions analysis.

Supplementary Figure S1: PLS-DA models and cross-validation parameters from the comparison between sorghum samples and no-sorghum samples.

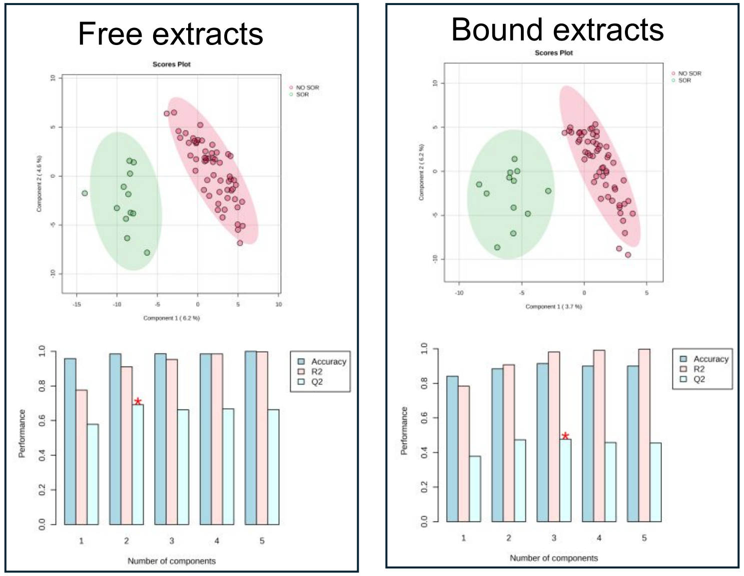

Supplement: Supplementary file 1 — jf4c01312_si_001.pdf [file jf4c01312_si_001.pdf]
